# Deciphering the mechanisms of cellular uptake of engineered nanoparticles by accurate evaluation of internalization using imaging flow cytometry

**DOI:** 10.1186/1743-8977-10-2

**Published:** 2013-02-06

**Authors:** Sandra Vranic, Nicole Boggetto, Vincent Contremoulins, Stéphane Mornet, Nora Reinhardt, Francelyne Marano, Armelle Baeza-Squiban, Sonja Boland

**Affiliations:** 1 Univ Paris Diderot, Sorbonne Paris Cité, Laboratory of Molecular and Cellular Responses to Xenobiotics, Unit of Functional and Adaptive Biology (BFA) EAC CNRS 4413, 5 rue Thomas Mann, Paris 75 013, France; 2 Institut Jacques Monod, Sorbonne Paris Cité, ImagoSeine BioImaging Core Facility, CNRS, UMR 7592, Univ Paris Diderot, Paris, France; 3 Institut de Chimie de la Matière Condensée de Bordeaux, UPR CNRS 9048, Université Bordeaux 1, 87 Avenue du Docteur A. Schweitzer, Pessac cedex F-33608, France

**Keywords:** Lung epithelial cells, ImageStream^X^, Endocytosis, Clathrin, Macropinocytosis, TiO_2_, SiO_2_

## Abstract

**Background:**

The uptake of nanoparticles (NPs) by cells remains to be better characterized in order to understand the mechanisms of potential NP toxicity as well as for a reliable risk assessment. Real NP uptake is still difficult to evaluate because of the adsorption of NPs on the cellular surface.

**Results:**

Here we used two approaches to distinguish adsorbed fluorescently labeled NPs from the internalized ones. The extracellular fluorescence was either quenched by Trypan Blue or the uptake was analyzed using imaging flow cytometry. We used this novel technique to define the inside of the cell to accurately study the uptake of fluorescently labeled (SiO_2_) and even non fluorescent but light diffracting NPs (TiO_2_). Time course, dose-dependence as well as the influence of surface charges on the uptake were shown in the pulmonary epithelial cell line NCI-H292. By setting up an integrative approach combining these flow cytometric analyses with confocal microscopy we deciphered the endocytic pathway involved in SiO_2_ NP uptake. Functional studies using energy depletion, pharmacological inhibitors, siRNA-clathrin heavy chain induced gene silencing and colocalization of NPs with proteins specific for different endocytic vesicles allowed us to determine macropinocytosis as the internalization pathway for SiO_2_ NPs in NCI-H292 cells.

**Conclusion:**

The integrative approach we propose here using the innovative imaging flow cytometry combined with confocal microscopy could be used to identify the physico-chemical characteristics of NPs involved in their uptake in view to redesign safe NPs.

## Background

The number of current and future applications of nanotechnology is growing exponentially, making possible the production of many innovative high-quality items as well as a lot of promising biomedical implementations. Great efforts are made in order to understand and estimate the risk for human health coming from the unintentional occupational or environmental exposure to nanoparticles (NPs). Biodistribution studies in animals have shown that NPs accumulate not only at the site of exposure but can reach the circulation by crossing the epithelial barriers [1]. In order to understand the mechanisms of transcytosis and potential NP toxicity to perform a reliable risk assessment it is of crucial importance to study NP uptake by epithelial cells. The capacity of NPs to pass the biological barriers has also let to foresee the application of nanotechnologies in the biomedical field for imaging and targeted drug delivery. The quantification of NP uptake is thus of crucial importance to evaluate the fate of tuned NPs in order to increase drug delivery by nanovectors, but also to develop NPs “safe by design”. Consequently, knowledge about the endocytic pathways involved in NP uptake is important to design safe NPs. Such issues are not simple since physico-chemical characteristics of NPs influence the interaction mechanisms leading to NP uptake [2-5].

Unfortunately, quantitative measurement of NP uptake still poses a technical challenge. It is still difficult to evaluate accurately real NP uptake because of the adsorption of NPs on the cell surface by interaction with the plasma membrane. This is one of the main limitations of spectroscopic methods that moreover are restricted to metallic particles. NPs can be observed inside the cells by transmission electron microscopy, but quantification of their uptake remains time and cost consuming [6]. NP uptake is generally quantified by flow cytometric analysis of fluorescently labeled NPs, [7] or of NPs that have the capacity to scatter the light [8]. In the literature, surprisingly, little attention has been paid to differentiate intracellular signal from the fluorescence or scattering due to NPs adsorbed on the cell surface, thus just providing a global signal.

In this article, we used fluorescent SiO_2_ NPs since they are easily labeled with different fluorochromes but have poor ability to scatter the light as well as non fluorescent TiO_2_ NPs as an example of NPs that are difficult to label with fluorochromes but that can well scatter the light. They are used in many industrial applications leading to the potential occupational exposures. Both NPs are also foreseen for nanomedical applications as drug delivery systems or in biomedical imaging. Our study was performed on a pulmonary epithelial cell line (NCI-H292) as the respiratory system is one of the most exposed to NPs.

We characterized the mechanisms of NP uptake by combining different approaches. Firstly, we focused on methods to distinguish adsorbed from internalized NPs. External fluorescence from adsorbed fluorescently labeled NPs was excluded by quenching using the vital dye Trypan Blue (TB) that is incapable of penetrating intact cell membranes. However, there is a whole panel of fluorochromes used to label NPs that cannot be quenched and this method did not allow analyzing fixed cells. Imaging flow cytometry, a newly developed technology, may allow not only determining the cellular localization of fluorescent, but also light diffracting, non fluorescent NPs [9]. This innovative technique permitted us to define the inside of the cell by eroding the cell borders to determine the ratio of internalization and to study NP uptake on a large number of cells.

These two flow cytometric techniques allowed us to study the time course, dose-dependency as well as the influence of surface charges on the uptake. They were also used to examine the endocytic pathways involved in NP uptake firstly by a rather broad approach using energy depletion and pharmacological inhibitors and secondly more specifically by using siRNA induced gene silencing. These functional studies were strengthened by confocal microscope observations used also for colocalization studies of NPs with proteins specific for different endocytic vesicles.

We provide evidence that ImageStream^X^, that associates flow cytometry and high resolution image analysis, allows an accurate evaluation of fluorescent or light diffracting NP uptake. By setting up an integrative approach to decipher the mechanisms of NP endocytosis, we showed that macropinocytosis is the predominant pathway of SiO_2_ NP internalization by NCI-H292 pulmonary epithelial cells and that TiO_2_ uptake is charge-dependent.

## Results and discussion

### Interactions of SiO_2_-NPs with NCI-H292 cells

Cells were treated with NPs at non cytotoxic concentrations (2.5 and 5 μg/cm^2^ for 50 nm-FITC-SiO_2_ NPs and 5 and 25 μg/cm^2^ for 100 nm-Por-SiO_2_ NPs) that were determined by WST-1 test (Additional file 1: Figures S1A and S1B in Supporting information).

**Figure 1:**
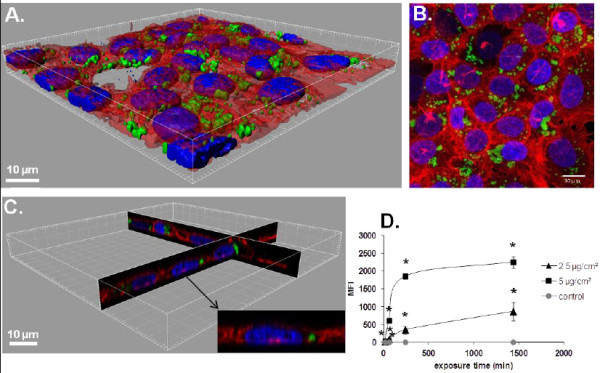
**Interaction of 50 nm-FITC-SiO**_**2 **_**with NCI-H292. A**. 3D reconstruction of a confocal analysis of the cells exposed to 50 nm-FITC-SiO_2_ NPs at 5 μg/cm^2^ for 24 h. Staining of the cells is as follows: Blue - DAPI-stained nuclei, Red – TRITC-phalloidin-stained actin filaments, Green – FITC-labelled SiO_2_ NPs. Scale bar shows 10 μm. **B**. The same field of the confocal image shown in the Figure 1A presented as a projection of all images acquired in the stack. **C**. 3D reconstruction of x,z and y,z-slices of the corresponding regions on the image 1A. The insert shows one selected representative cell and **D**. Cells were exposed to different concentrations of NPs at indicated time points, followed by FCM analysis of median fluorescence intensity (MFI) of at least 10.000 cells. Results are represented as mean MFI value ± SD, n=3 of one out of 3 independent experiments. Data were analyzed by ANOVA, followed by Bonferroni post hoc test. * significantly different from previous time point, *p* < 0.05.

**Figure 2:**
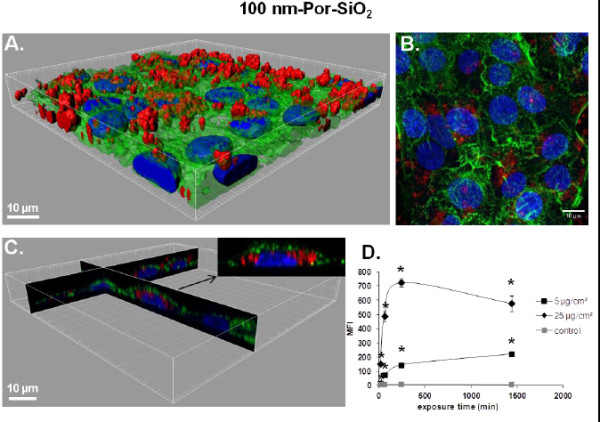
**Interaction of 100 nm-Por-SiO**_**2 **_**NPs with NCI-H292. A**. 3D reconstruction of a confocal analysis of cells exposed to 100 nm-Por-SiO_2_ NPs at 25 μg/cm^2^ for 24 h. Staining of the cells is as follows: Blue - DAPI-stained nuclei, Green - FITC-phalloidin-stained actin filaments, Red - Porphyrine-labelled SiO_2_ particles. Scale bar shows 10 μm. **B**. The same field of the confocal image shown in the Figure 2A presented as a projection of all images acquired in the stack. **C**. 3D reconstruction of x,z and y,z-slices of the corresponding regions of the image 2A. The insert shows one selected representative cell and **D**. Cells were exposed to different concentrations of NPs at indicated time points, followed by FCM analysis of median fluorescence intensity (MFI) of at least 10.000 cells. Results are represented as mean MFI value ± SD, n=3 of one out of 3 independent experiments. Data were analyzed by ANOVA, followed by Bonferroni post hoc test. * significantly different from previous time point, *p* < 0.05.

As shown by flow cytometry (FCM), 50 nm-FITC-SiO_2_ treated cells exhibited a time and dose dependent increase of their median fluorescence intensity (MFI). It was significantly different from the control already after 15 min of exposure for both tested concentrations (Figure 1D). For 100 nm-Por-SiO_2_ treated cells MFI values were significantly different from the control after 15 min of exposure to 25 μg/cm^2^ and from 1 h for 5 μg/cm^2^ compared to the MFI of non-treated cells, (Figure 2D). As observed on three-dimensional (3D) reconstructions of z-slices obtained by confocal microscopy (Figures 1C and 2C), NPs were found inside the cells but they could also be observed on the cell surface. Observation of treated cultures revealed that 50 nm-FITC-SiO_2_ were almost entirely internalized after 24 h suggesting that the MFI measured by FCM mainly reflected internalized NPs (Figure 1A-C). Conversely, for 100 nm-Por-SiO_2_ confocal imaging revealed a high adsorption of NPs on membranes suggesting the inaccuracy of FCM analysis to quantitatively appreciate NP uptake (Figure 2A-C). The interpretation of results from FCM has to be performed very carefully as thorough rinsing made after incubation with NPs may not be sufficient to remove NPs that are firmly adsorbed onto cell membranes as we have shown here for 100 nm-Por-SiO_2_ NPs.

Comparison with 50 nm-FITC-SiO_2_ NPs let us to conclude that the adsorption of NPs on the cell surface is NP dependent and should be carefully verified before interpretation of the results obtained by FCM. Numerous studies have shown interactions of fluorescent NPs with different cell lines by FCM and/or confocal microscopy [10-12] and adsorption of some SiO_2_ NPs on the cell surface has also been reported, [13-15] but have rarely been taken into account for the quantification of their uptake. Confocal microscopy permits to localize NPs, while FCM gives statistical quantification of the interactions by evaluation of MFI of the treated cells. This quantification allows a relative comparison between treatment conditions. By the analysis of NP-cell interactions with these two techniques in parallel we showed that this global analysis of cell fluorescence by FCM is not suitable to quantify the uptake in case of adsorbed NPs.

### Elimination of the fluorescent signal from adsorbed NPs by quenching

**Figure 3:**
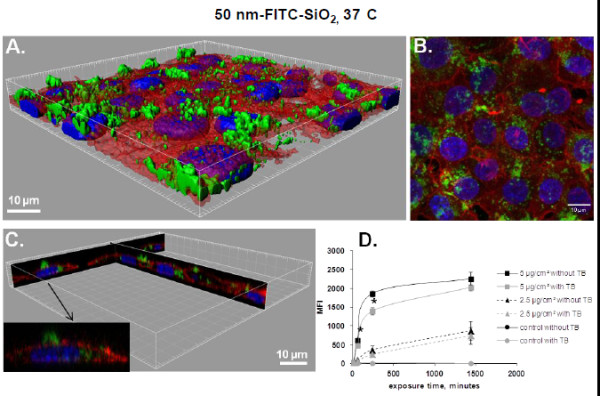
**Determination of 50 nm-FITC-SiO**_**2 **_**uptake in NCI-H292 cells by flow cytometry and confocal microscopy. A**. 3D reconstruction of the confocal analysis of cells exposed to 50 nm-FITC-SiO_2_ NPs at 5 μg/cm^2^ for 4 h at 37°C. Staining of the cells is as follows: Blue - DAPI-stained nuclei, Red – TRITC-phalloidin-stained actin filaments, Green – FITC-labelled SiO_2_ NPs. Scale bar shows 10 μm. **B**. The same field of the confocal image shown in the Figure 3A presented as a projection of all images acquired in the stack. **C**. 3D reconstruction of x,z and y,z-slices of the corresponding regions of the image 3A. The insert shows one selected representative cell and **D**. Cells were incubated with 50 nm-FITC-SiO_2_ at 37°C at indicated concentrations. Median fluorescence intensity (MFI) of at least 10.000 cells was analysed by FCM without or with 0.11% TB added just before FCM analysis. Results are represented as mean MFI value ± SD, n=3 of one out of 3 independent experiments. Data were analyzed by ANOVA, followed by Bonferroni post hoc test. * significantly different after TB addition, *p* < 0.05.

To accurately quantify NP uptake, the analysis of cellular MFI by FCM can be improved using stains able to quench the fluorescence that comes from the outside of the cells. Trypan Blue (TB) has been demonstrated to quench the fluorescence of FITC-labeled compounds when it comes in close contact with them [16-18]. According to its physico–chemical properties TB cannot pass intact membranes of viable cells and is therefore unable to quench intracellular fluorescence [19]. This was verified using a mitochondrial marker 3, 3^′^-Dihexyloxacarbocyanine iodide (DiOC_6_(3)), exhibiting green fluorescence that is not quenched when cells are incubated with TB (Additional file 1: Supporting Figure S2). Figure 3D shows the time course of MFI of 50 nm-FITC-SiO_2_ treated cells analyzed by FCM before and after adding TB. The MFI of cells exposed for 4 h to 5 μg/cm^2^ diminishes by 25% after TB addition, suggesting that 75% of the NPs were internalized. After 24 h of exposure NPs are almost entirely internalized (90%), confirming observations by confocal microscopy (Figures 1A-C and 3A-C). The same trend was observed at the lower dose (2.5 μg/cm^2^): 70% of the NPs were internalized after 4 h and 85% after 24 h. Thus the elimination of the adsorbed part of NPs allows showing that internalization is time and dose dependent.

**Figure 4:**
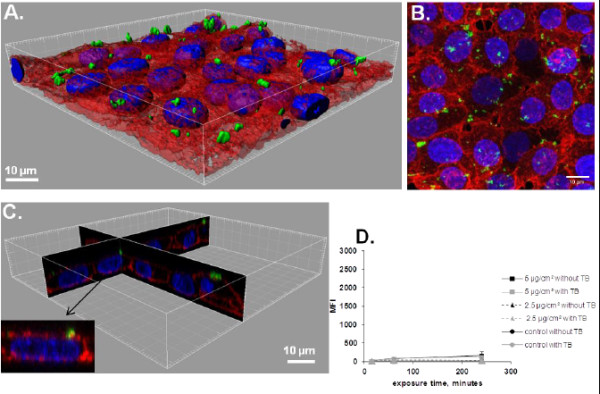
**Determination of 50 nm-FITC-SiO**_**2 **_**uptake in NCI-H292 cells by flow cytometry and confocal microscopy. A**. 3D reconstruction of a confocal analysis of cells exposed to 50 nm-FITC-SiO_2_ NPs at 5 μg/cm^2^ for 4 h at 4°C. Staining of the cells is as follows: Blue - DAPI-stained nuclei, Red – TRITC-phalloidin-stained actin filaments, Green – FITC-labelled SiO_2_ NPs. Scale bar shows 10 μm. **B**. The same field of the confocal image shown in the Figure 4A presented as a projection of all images acquired in the stack. **C**. 3D reconstruction of x,z and y,z-slices of the corresponding regions of the image 4A. The insert shows one selected representative cell and **D**. Cells were pre-incubated for 30 min at 4°C, and subsequently exposed to 50 nm-FITC-SiO_2_ at 4°C. Median fluorescence intensity (MFI) of at least 10.000 cells was analysed by FCM without or with 0.11% TB added just before FCM analysis. Results are represented as mean MFI value ± SD, n=3 of one out of 3 independent experiments.

To validate this method of quantifying the amount of internalized NPs, cells were treated with NPs at 4°C. At this temperature, energy dependent uptake as well as passive diffusion are blocked due to the rigidity of the membrane that does not enable passive internalization to take place. Indeed, on the confocal images and 3D reconstructions there were no NPs observed inside the cells exposed for 4 h to 50 nm-FITC-SiO_2_ at 4°C (Figure 4A-C) conversely to cells exposed at 37°C (Figure 3A-C) The MFI values at 4°C (Figure 4D) were much lower than at 37°C (Figure 3D). The MFI was however still slightly different compared to control cells (Figure 4D) suggesting that some NPs could have been internalized during the trypsination step required for FCM analysis or that TB was unable to completely quench FITC fluorescence from the surface as it has already been described [20]. Furthermore, the efficacy of TB is restricted to some wavelengths. Indeed, we observed that TB was inefficient in quenching porphyrine labeled 100 nm-SiO_2_-NPs. Due to these limitations (efficiency of quenching, wavelength limitations and impossibility to study fixed cells) we looked for another technique to quantify the uptake of NPs.

### Elimination of the fluorescent signal from adsorbed NPs by Imaging flow cytometry

**Figure 5:**
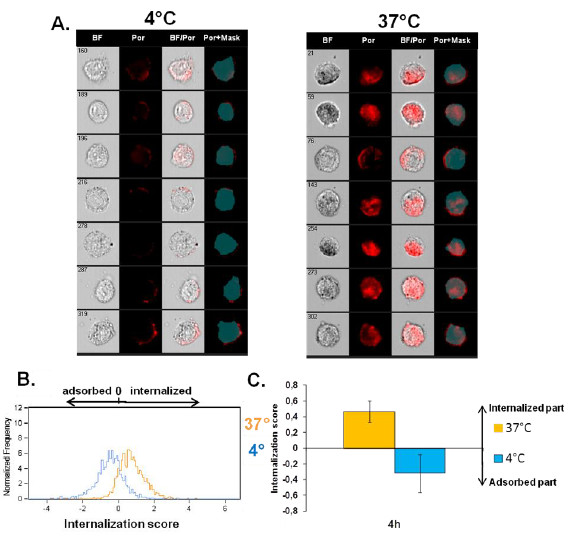
**Determination of 100 nm-Por-SiO**_**2 **_**uptake by NCI-H292 cells by imaging flow cytometry. A**. Representative images captured by the Amnis ImageStream^X^ Flow Cytometer of cells treated with 100 nm-Por-SiO_2_ for 4 h at 37°C or 4°C. First column shows brightfield (BF) images of the cells, second column shows images of fluorescence of porphyrine (Por), third column shows fluorescence merged with the brightfield images of the cells (BF/Por) and forth column shows the applied mask eroded for 3 μm and porphyrine fluorescence (Por+Mask). B. and C. Internalization score (IS) calculated by Amnis IDEAS software: distribution of IS of at least 500 cells treated for 4 h at 37 °C or 4°C (**B**.), and corresponding mean value of IS ± SD of six independent experiments (**C**.).

100 nm-Por-SiO_2_ were tightly attached to the cell membrane after thorough rinsing (Figure 2A-C) and fluorescence from adsorbed NPs could not be eliminated efficiently by classic fluorescence quenchers. We thus used ImageStream^X^ platform which is an imaging flow cytometer that combines the speed, statistical power and fluorescence sensitivity of flow cytometry with the functional performance of high resolution microscopy. Powerful image analysis software allows quantification of the fluorescence at different cellular localizations. A mask representing the whole cell was defined by the bright-field image, and an internal mask was defined by eroding the whole cell mask by 3 μm (6 pixels) in order to eliminate the fluorescent signal coming from NPs attached to the cell surface thus measuring only the internalized part. Figure 5A shows the difference in NP uptake in cells treated for 4 h with NPs at 4°C compared to 37°C. Images taken by the ImageStream^X^ imaging flow cytometer show that at 4°C NPs were located predominantly on the cell surface whereas after treatment at 37°C, NPs were found inside the cells as well as on the cell surface. This localization of NPs in cells exposed at 4°C was confirmed by confocal microscopy (Additional file 1: Supporting Figure S3). Comparison of the fluorescence detected inside the eroded mask with whole cell fluorescence enabled the determination of internalization score (IS) that represents the ratio of fluorescence intensity inside the cell to the total fluorescence intensity of the cell. Calculation of IS has been explained in Supporting Information, (Section 4). A positive value of IS corresponds to a cell with mostly internalized NPs whereas a negative IS corresponds to a cell with mostly surface-associated NPs. If the IS value is around 0 there is an equal amount of NPs that are adsorbed and internalized. Distribution curves of the IS values on the Figure 5B and the mean IS on Figure 5C show that most cells treated at 4°C had a negative IS indicating NP adsorption on the cell surface. For the cells treated at 37°C, the IS was positive, showing that the majority of the cells had internalized NPs confirming the observations on the corresponding images in Figure 5A. These results indicate that imaging flow cytometry analysis using a cell mask eroded for 3 μm is suitable to distinguish adsorbed from internalized NPs. This approach was also validated by analyzing the uptake of 50 nm-FITC-SiO_2_ NPs (Additional file 1: Supporting Figure S4) as the results were similar to TB quenching using classic FCM (Figure 3D).

**Figure 6:**
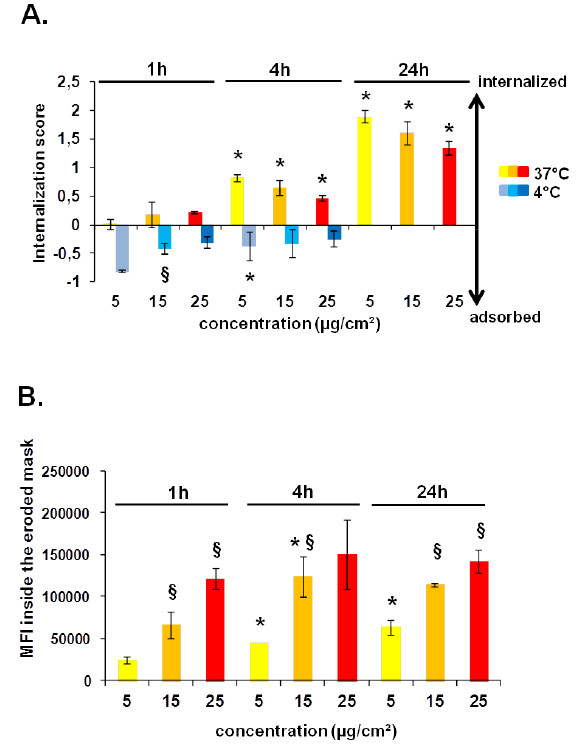
**Internalization of 100 nm-Por-SiO**_**2 **_**by NCI-H292 cells analyzed by imaging flow cytometry. A**. Internalization score obtained using a mask eroded for 3 μm after treatment with 100 nm-Por-SiO_2_ at 37 °C or 4°C, at different concentrations and at different time points. **B**. Mean fluorescence intensity (MFI) inside the mask eroded for 3 μm for cells treated with 100 nm-Por-SiO_2_ at different time points and at different concentrations. Values are expressed as mean IS (**A**.) or mean value of MFI (**B**.) ± SD of six independent experiments analyzing at least 500 cells. Data were analyzed by ANOVA, followed by Bonferroni post hoc test. * significantly different from previous time point, *p* < 0.05. ^§^significantly different from lower concentration, p < 0.05.

Applying this approach, we were able to show that the IS of 100 nm-Por-SiO_2_ increased with the duration of the exposure and that at 4°C NPs were predominantly adsorbed on the cell surface for all tested time points and concentrations (Figure 6A). In addition we showed that cells treated at lower concentrations had a higher IS suggesting that NPs could be better taken up at low doses. Indeed, as the IS is the ratio of internal fluorescence compared to total fluorescence, it indicates the uptake efficiency of bound NPs. To accurately compare the uptake of different concentrations of NPs we determined the MFI inside the cell mask eroded for 3 μm. As shown in Figure 6B the MFI due to internalized 100 nm-Por-SiO_2_ increased with dose and time of exposure. At the highest dose, the increase of MFI over time was low suggesting that from 1 h, epithelial cells have reached their maximal endocytic potential. This might be due to the saturation of uptake mechanisms as the IS also decreased with increasing concentrations after 4 and 24 h of treatment. Conversely, at lower doses NPs continued to be efficiently taken up until 24 h of treatment.

Using these two parameters (IS and MFI inside the eroded cell mask) analyzed by ImageStream^X^ Ideas software we have obtained important information about NP uptake. Comparing IS values after treatment at 37° and 4°C allowed us to define an eroded cell mask to distinguish adsorbed from internalized part. Using MFI inside the eroded cell mask we could establish the time and dose-dependency of SiO_2_ NP internalization. Combining these two parameters allowed us to demonstrate the saturation of endocytic mechanisms of NPs.

### Mechanisms of NP uptake: energy dependence

**Figure 7:**
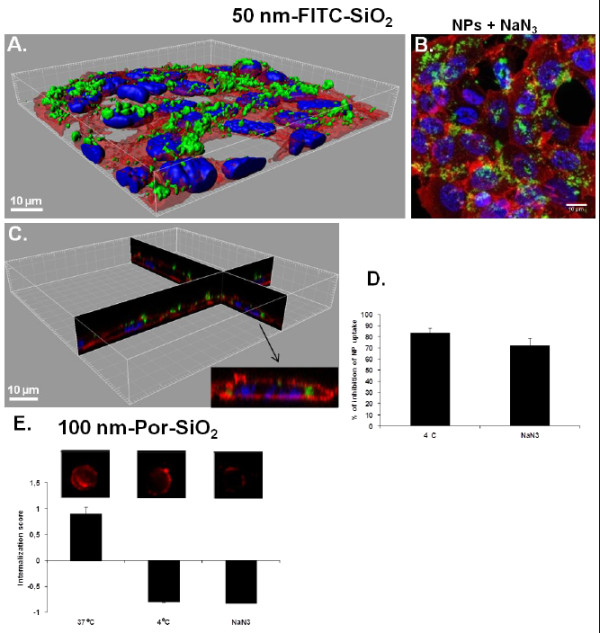
**Energy dependence of NP internalization. A**. 3D reconstruction of a confocal analysis of cells exposed to NPs and NaN_3_ for 4 h. Staining of the cells is as follows: Blue - DAPI-stained nuclei, Red – TRITC-phalloidin-stained actin filaments, Green – FITC-labelled SiO_2_ NPs. Scale bar shows 10 μm. **B**. The same field of the confocal image shown in the Figure 7A presented as a projection of all images acquired in the stack. **C**. 3D reconstruction of x,z and y,z-slices of the corresponding regions of the image 7A. and D and E. Cells were either pre-incubated at 37°C with 100 mM of NaN_3_ for 30 min, or incubated at 4°C before being exposed to NPs at 5 μg/cm^2^ for 4 h. Quantification of 50 nm-FITC–SiO_2_ uptake was performed by flow cytometry after addition of TB (**D**). Analysis of the internalization of 100 nm-Por-SiO_2_ NPs was performed by imaging flow cytometry using a mask eroded for 3 μm. Representative fluorescence images of cells are shown. Results are expressed as mean value of the percentage of inhibition of NP uptake (**D**.) or mean value of Internalization score (**E**.) ± SD, n=3 at least.

In order to determine whether NP uptake was an active or passive process, cells were energy depleted using sodium azide (NaN_3_) that is known to inhibit the respiratory chain in the mitochondria, thus impairing the production of ATP in the cell and consequently the active uptake. Sodium azide inhibited the uptake of 50 nm-FITC-SiO_2_ NPs up to 76% (Figure 7D). We compared inhibition of uptake by NaN_3_ to the inhibition at 4°C that prevents not only active uptake but also the passive uptake by increasing the rigidity of the plasma membrane. At 4°C the percentage of inhibition is higher (85%) than for the cells treated with NaN_3_ (76%), suggesting that some 50 nm-FITC-SiO_2_ NPs may enter by passive diffusion. This was confirmed by confocal microscopy showing that NPs were not only on the cell surface but also inside the cells after treatment with sodium azide (Figure 7A-C) in contrast to treatment at 4°C (Figure 4A-C). Internalization of NPs by a non endocytic pathway has already been proposed [21,22] and NPs have been observed in red blood cells that lack endocytic properties [22]. This passive internalization by red blood cells has been shown to involve adsorption of NPs on the cell surface and strong local membrane deformations leading to internalization [23]. Using large unilamelar liposomes as a simplified model of lipid membrane, passive uptake of silica NPs has further been explained by lipid membrane spreading around NP surfaces by a mechanism that involves adhesion and bending energies [24].

Concerning 100 nm-Por-SiO_2_ NPs, energy depletion with NaN_3_ induced the same decrease of the IS as the treatment at 4°C, suggesting complete inhibition of the uptake by NaN_3_ and thus no passive diffusion (Figure 7E). This is confirmed by the corresponding images obtained by the imaging flow cytometer (Figure 7E). The absence of passive diffusion of 100 nm-Por-SiO_2_ NPs can be explained by the bigger size of the NPs as it has already been shown that passive uptake of SiO_2_ is size dependent [23]. Furthermore these NPs form aggregates that may not permit passive processes to occur.

### Effect of pharmacological inhibitors

**Figure 8:**
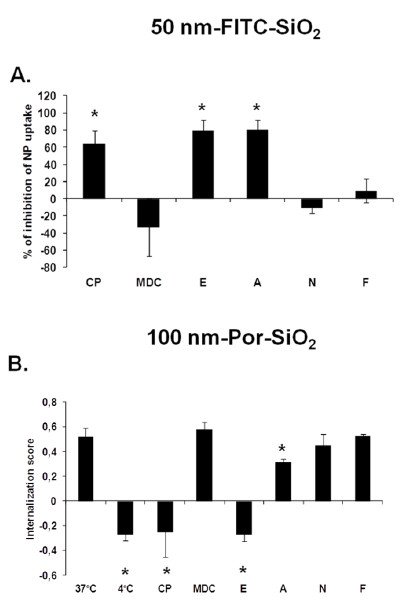
**Effect of pharmacological inhibitors on the uptake of 50 nm-FITC-SiO**_**2 **_**and 100 nm-Por-SiO**_**2 **_**NPs.** Cells were pre-treated with inhibitors of main endocytotic pathways: Chlorpromazine (CP) at 25 μM, Monodansylcadaverine (MDC) at 75 μM, EIPA (E) at 75 μM, Amiloride (**A**) at 1.5 mM, Nystatin (N) at 75 μM and Filipin (F) at 4.5 μM for 30 min and then exposed to A.: 50 nm-FITC-SiO_2_ NPs at 5 μg/cm^2^ and inhibitors for 3.5 h. Quantification of the internalization was performed by flow cytometry, after addition of TB. Results are expressed as mean percentage of inhibition of NP uptake in cells not treated with inhibitors ± SD, n = 6–18. **B**.: 100 nm-Por-SiO_2_ NPs at 15 μg/cm^2^ and inhibitors or at 4°C for 3.5 h. Analysis of the internalization was performed by imaging flow cytometry using a mask eroded for 3 μm. Results are expressed as mean Internalization score ± SD of four independent experiments. Data were analyzed by ANOVA, followed by Bonferroni post hoc test. * significantly different from treatment with NPs at 37°C in the absence of inhibitors, *p* < 0.05.

As the results with NaN_3_ showed that SiO_2_ uptake is mainly due to an active process, we studied which endocytic mechanism is implied. Cells were treated with inhibitors for the three major endocytic pathways: chlorpromazine (CP) and monodansylcadaverine (MDC) for clathrin dependent endocytosis, EIPA (E) and amiloride (A) for macropinocytosis and nystatin (N) and filipin (F) for caveolae dependent endocytosis. Mechanism of action of pharmacological inhibitors has been explained in Supporting Information (Section 6). Cells were treated with inhibitors at non cytotoxic concentrations determined by WST-1 test as well as by PI staining of the cells treated with inhibitors (Additional file 1: Supporting Figures S5A and S5B). When using endocytic inhibitors it is important to ensure that they do not affect the actin cytoskeleton [25]. Reorganization of the actin filaments may impact the uptake processes even if they do not directly involve actin as this reorganization may alter the function of various plasma membrane proteins, [26] thereby confounding the data and leading to multiple effects occurring simultaneously. We thus first verified that actin filaments were intact after the treatment with different inhibitors (confocal images shown in Additional file 1: Supporting Figure S6). Quantification of the uptake using TB quenching showed that for 50 nm-FITC-SiO_2_ NPs the highest inhibition of uptake could be achieved with two macropinocytosis inhibitors E (84%) and A (80%), and the clathrin dependent endocytosis inhibitor CP (74%) (Figure 8A). The second inhibitor used for clathrin dependent endocytic pathway (MDC) did not prevent the uptake and neither did N and F that were used as inhibitors of caveolae dependent endocytosis.

Figure 8B shows the inhibition of uptake of 100 nm-Por-SiO_2_ NPs studied by imaging flow cytometry. Negative values of the internalization score were obtained for CP, E and A suggesting low internalization after treatment with these inhibitors. After treatment with CP and E, the IS was similar to the IS observed at 4°C indicating complete inhibition of NPs internalization with these inhibitors. The second inhibitor for clathrin dependent endocytosis (MDC) did not prevent NP uptake neither did the caveolae dependent endocytosis inhibitors (N and F).

These results using pharmacological inhibitors indicate that both types of SiO_2_ NPs seem to be taken up by macropinocytosis and clathrin dependent endocytosis in NCI-H292 cells. However, for both SiO_2_ NPs studied we observed contradictory results with the two clathrin dependent endocytosis inhibitors as MDC had no effect on their uptake. Utilization of pharmacological inhibitors is a common approach for studying endocytic pathways, hence, their efficacy has been largely questioned [25,27]. They can also lack specificity for defined pathways [25] and are shown to be cell type dependent [27]. Balance should be found between the concentration of inhibitor high enough to inhibit endocytosis but not to induce cytotoxicity. For 100 nm-Por-SiO_2_ NPs the efficacy of Amiloride was lower than that of EIPA. This may be explained by different potencies of these two inhibitors as Amiloride blocks macropinocytosis when used at millimolar concentrations, [28] whereas EIPA is effective in the range of 50–100 μ*M*[29,30].

### Effect of siRNA induced gene silencing

**Figure 9:**
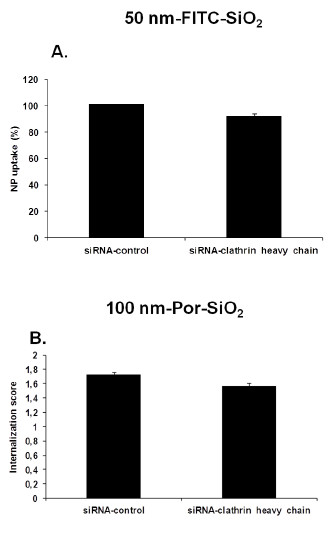
**Effect of siRNA induced gene silencing of clathrin heavy chain on SiO**_**2 **_**NP uptake.** Cells were treated with siRNA-control or siRNA-clathrin heavy chain for 72 h before treating with **A**.: 50 nm-FITC-SiO_2_ NPs for 3.5 h. Quantification of the internalization was performed by flow cytometry after addition of TB. Results are expressed as percentage of NP uptake compared to uptake in the siRNA-control treated cells, mean percentage ± SD, *n* = 9 **B**.: 15 μg/cm^2^ of 100 nm-Por-SiO_2_ NPs for 3.5 h. Analysis of the internalization was performed by imaging flow cytometry using a mask eroded for 3 μm. Results are expressed as mean Internalization score ± SD of three independent experiments.

Considering the opposite effect of two inhibitors for clathrin dependent endocytosis, the implication of this pathway in NP uptake was further investigated using a more specific approach consisting in siRNA induced silencing of clathrin heavy chain (CHC) protein. Depletion of clathrin heavy chain expression after 72 h of treatment was verified by Western blot and confocal microscopy (Additional file 1: Supporting Figures S7 and S8). Figure 9A shows no significant difference between internalization of 50 nm-SiO_2_-FITC NPs in the cells treated with siRNA-control compared to those treated with siRNA-clathrin heavy chain. Figure 9B shows that similar results were obtained for 100 nm-Por-SiO_2_ NPs. Altogether these results underline that bronchial epithelial cells did not internalize 50 and 100 nm NPs by clathrin dependent endocytosis. Results obtained using clathrin dependent endocytosis inhibitor CP can be explained by the non specificity of this inhibitor that has already been reported [25]. Side effects of CP can be due to its amphipathic property increasing lipid fluidity within the plasma membrane [31] and preventing theformation of large membrane invaginations [32]. In addition CP has been reported to inhibit phospholipase C [33] an important regulator of actin dynamics [34] and macropinocytosis [35,36].

### Colocalization study

In order to confirm that SiO_2_ NPs uptake predominantly involves formation of macropinosomes and not clathrin or caveolae coated vesicles, we studied the colocalization of NPs with proteins that are specific for these vesicles. Cells were treated with SiO_2_ NPs during 4 h and observed for colocalization with clathrin heavy chain, caveolin-1 and sorting nexin-5 which is involved in macropinocytosis [37,38].

**Figure 10:**
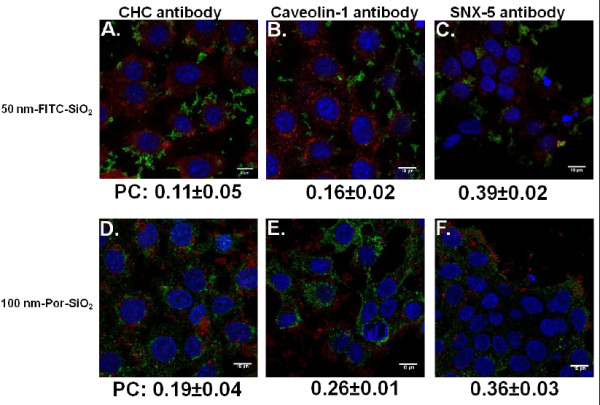
**Colocalization of SiO**_**2 **_**NPs with proteins specific for different endocytotic vesicles analyzed by confocal microscopy.** Cells treated with SiO_2_ NPs for 4 h were fixed and immunolabelled with Clathrin Heavy Chain antibody (CHC, 8A and 8D), Caveolin-1 antibody (8B and 8E) and Sorting Nexin-5 antibody (SNX-5, 8C and 8F). **A**, **B** and **C**: 50 nm-FITC-SiO_2_ NPs. Staining is as follows: Blue – DAPI-stained nuclei, Red –CHC, SNX-5, and caveolin-1 labelling, Green – FITC-labelled SiO_2_ NPs. **D**, **E** and **F**: 100 nm-porphyrine-SiO_2_ NPs. Staining is as follows: Blue - DAPI-stained nuclei, Green – CHC, SNX-5 and caveolin-1 labelling, Red – Porphyrine-labelled SiO_2_ NPs. Scale bar shows 10 μm. Pearson’s coefficients were calculated and expressed as mean value ± SD for at least 3 images obtained in three independent experiments.

Colocalization was studied by confocal microscopy and then quantified by Pearson’s coefficient that could vary from +1 in the case of a perfect positive linear relationship (correlation) to −1 in the case of a perfect negative linear relationship (anticorrelation), with zero if no relationship (uncorrelated). 50 nm-FITC-SiO_2_ NPs had the highest coefficient of colocalization with the macropinosome marking using SNX-5 antibody (0.39±0.02) while the Pearson’s coefficients with clathrin heavy chain (0.11±0.05) and caveolin-1 (0.16±0.02) were lower (Figure 10A, B, C). Similar results were obtained for 100 nm-Por-SiO_2_ NPs showing the highest colocalization coefficient with SNX-5 (0.36±0.03) while values for clathrin heavy chain (0.19±0.04) and caveolin-1 (0.26±0.01) were lower (Figure 10D, E, F). The relatively low Pearson’s coefficients could be explained by the short half-life of coated vesicles. The specific proteins involved in vesicle formation will rapidly dissociate and the endocytic vesicles will evolve into early endosomes that mature to late endosomes and lysosomes where the NPs may end up. Immunohistochemistry using the lysosomal marker LAMP (Additional file 1: Supporting Figure S9) shows indeed that after 4 and 24 h of exposure, NPs localized in the lysosomes. This also indicates that they were transported by the vesicular system inside the cell that requires energy confirming results obtained with NaN_3_.

These different approaches combining confocal microscopy and imaging flow cytometry using inhibitors or siRNA silencing reveal that both SiO_2_ NPs are taken up by macropinocytosis. SiO_2_ NP uptake has also been shown to be independent from clathrin and caveolae dependent endocytosis in A549 cells [7,39]. However, Chung et al. analyzed mechanisms of silica NPs uptake in mesenchymal cells after inhibition of clathrin dependent pathway with Phenylarsine Oxide (PAO) and of macropinocytosis by Cytochalasin D and found that the uptake can occur not only by actin dependent but also by clathrin dependent endocytosis [40]. However PAO is not specific for the clathrin pathways and has also been shown to inhibit macropinocytosis in adipocytes [41] and phagocytosis in mast cells [42]. Meng et al. have demonstrated that macropinocytosis is implied in the internalization of silica NPs with high aspect ratio by small GTPase-dependent macropinocytosis mechanism [43]. These studies support our conclusion that studies using inhibitors have to be analyzed with caution.

On the other hand these differences between studies on SiO_2_ uptake mechanisms may also be due to size variations of aggregates formed. In the study of Gyenge et al. TEM revealed that single silica NPs were most likely internalized by clathrin coated pits, while larger aggregates of NPs were internalized by membrane ruffling characteristic for macropinocytosis [13]. These findings are in agreement with the size of our NP aggregates evaluated by DLS analysis showing 1μm and 300 nm aggregates for 100 nm-Por-SiO_2_ NPs and 50 nm- FITC-SiO_2_ NPs respectively. Clathrin coated vesicles have indeed diameters of about 120 nm while macropinocytotic vesicles can accommodate molecules of more than 1 μm size. Although recent publications emphasize the size of NPs as a critical factor for entering the respective uptake pathways, [44] it cannot be applied as a general rule as in some studies it has been shown that larger NPs (500 nm) can be taken up exclusively by caveolae-dependent pathway while these vesicles are not bigger than 80 nm [45].

### Applying of the imaging flow cytometry to study internalization of non fluorescent NPs

**Figure 11:**
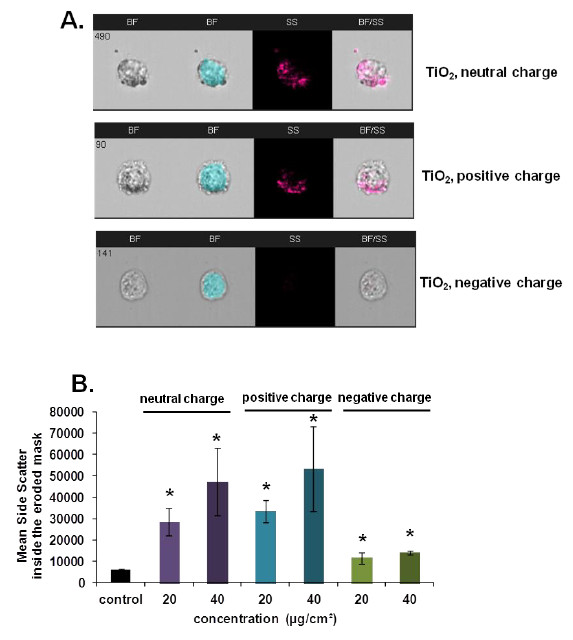
**Internalization of TiO**_**2 **_**NPs by NCI-H292 cells analyzed by imaging flow cytometry. A**. Representative images captured by Amnis ImageStream^X^ Flow Cytometer of cells treated with neutral, positively or negatively charged TiO_2_ NPs for 4 h at 20 and 40 μg/cm^2^. First column shows cells in the brightfield (BF), second shows the mask eroded for 3 μm, third column shows the signal of the side scatter (SS) and forth column shows images of the brightfield merged with the side scatter signal (BF/SS). **B**. Intensity of the side scatter signal inside the mask eroded for 3 μm. Results are expressed as mean value ± SD of three independent experiments analyzing at least 500 cells. Data were analyzed by ANOVA followed by Bonferroni post hoc test. * significantly different from control, *p* < 0.05.

We intended to extend the method we developed with ImageStream^X^ imaging flow cytometry for fluorescently labeled NPs to unlabeled but light scattering NPs. We applied the eroded cell mask that defines the inside of the cell to exclude the side scatter signal from NPs adsorbed on the surface of the cell. We compared the uptake of different TiO_2_ NPs as they have the ability to produce a high side scatter signal contrarily to SiO_2_ NPs. Non cytotoxic concentrations of TiO_2_ were determined by WST-1 test (Additional file 1: Supporting Figure S10). Figure 11A shows that after 4 h of treatment at 40 μg/cm^2^ NPs are present inside the cells as well as on the cell surface. The intensity of the side scatter within the eroded cell mask increased according to the dose of treatment (Figure 11B) showing that NP internalization is dose dependent. Furthermore the uptake varies according to the surface charge of TiO_2_ NPs. Neutral and positively charged TiO_2_ NPs are more internalized than negatively charged NPs. This could be explained by the negative charge of the cell membrane that favors binding of the positively charged NPs to its surface while the negatively charged NPs are being repulsed.

Until now internalization of non fluorescent NPs has been based on global estimations of side scatter values obtained by flow cytometry, [8,46] an approach that cannot discriminate between adsorbed and internalized NPs. The same stands for studying internalization by different quantitative spectroscopic or chemical detection methods [47]. On the other hand estimations obtained by electron microscopy remain complex to perform, time consuming and rather qualitative [6]. The approach using ImageStream^X^ platform we have developed here for the first time can be helpful in fulfilling critical need for the uptake assessment of TiO_2_ or other non fluorescent but light scattering NPs and can be implemented in a battery of screening tests, especially as the cytotoxic effects of NPs could be closely linked to their internalization. The efficient inhibition of NP uptake assessed with ImageStream^X^ may allow determining whether adverse effects are dependent on NP internalization.

## Conclusions

An accurate evaluation of NP uptake is necessary for a better understanding of NP toxicity. We have demonstrated that identification of the mechanism of NP uptake has to be studied by different approaches as all of them have their limitations. One of the most complete approaches to study NP uptake is a combination of confocal microscopy for determining the localization of NPs and FCM for a statistically reliable quantification of uptake. However, to estimate the internalized fraction of applied NPs it should be taken into consideration that certain nanomaterials tend to firmly adsorb onto cellular membranes. This highlights the importance to discriminate between internalized and extracellular particles when assessing particle uptake. Using ImageStream^X^, which combines FCM with high resolution microscopy, we propose a new method to distinguish between adsorbed and internalized NPs that can be applied not only to fluorescent NPs, but also to NPs that scatter light.

Applying this method we have shown that the uptake of SiO_2_ NPs by NCI-H292 cells is a time and dose-dependent process that can be saturated. Moreover in combination with qualitative observations by confocal microscopy, we have demonstrated that this internalization is predominantly an energy dependent process but that 50 nm-FITC-SiO_2_ can also be taken up by passive diffusion. The use of pharmacological inhibitors of endocytosis suggests that SiO_2_ NPs are taken up by macropinocytosis and the clathrin dependent pathway. However, using more specific siRNA-clathrin heavy chain silencing, definitive evidence was found that clathrin mediated endocytosis is not involved in the uptake of SiO_2_ NPs by NCI-H292 cells. This also underlines the importance of a cautious interpretation of results obtained with pharmacological inhibitors. Experiments of NP colocalization with proteins specific for vesicles of each of three principal endocytic pathways further confirmed the involvement of macropinocytosis in SiO_2_ NP uptake by NCI-H292. Adapting imaging FCM to non fluorescent TiO_2_ NPs allowed us to demonstrate the influence of surface charges on their uptake which is reduced by the presence of negative charges.

Our study is the first to propose an integrative approach to characterize NP uptake by combining functional studies with confocal microscopy and the imaging flow cytometer ImageStream^X^. This powerful detection system allows distinguishing between adsorbed and internalized fluorescent as well as non-fluorescent NPs. ImageStream^X^ provides thus a reliable statistical quantification of the intracellular signal and the ratio of internalization based on the analysis of a large population of living or even fixed cells. This integrative approach could be used to identify the physico-chemical characteristics of NPs involved in their uptake and the role of the internalization in their adverse effects in view to redesign safe NPs.

## Methods

### Nanomaterial synthesis

Fluorescent SiO_2_ NPs of two sizes (50 and 100 nm) were synthesized. Fluorochromes were incorporated inside the NP core during synthesis. 50 nm-FITC-SiO_2_-NPs were labeled with fluorescein isothiocyanate (FITC) that is characterized by an excitation wavelength at 488 nm and maximum emission wavelength at 520 nm. The NPs were synthesized as described in Supporting Information (section 12). 100 nm-Por-SiO_2_-NPs were synthesised as already described [48]. They were coupled with 5, 10, 15, 20 -Tetrakis-(1-methyl-4pyridino) porphyrine tetra (toluene-4-sulfonate) or shortly porphyrine characterized by an excitation wavelength at 422 nm and maximum emission wavelengths at 666 nm and 716 nm. Non fluorescent TiO_2_ NPs of 10 nm size with neutral, positive and negative surface charges were synthesized as described previously [49].

### Nanomaterial characterization (TEM, DLS, fluorescence excitation/emission spectrums)

Morphology and aggregation of SiO_2_ NPs were verified by Transmission Electron Microscopy (TEM, JEOL 1200 EXII (OXFORD LINK ISIS 300)) as well as by Dynamic Light Scattering (DLS) analysis in RPMI 1640 cell culture medium (Life Technologies). For DLS characterization, NPs were prepared at the highest concentrations used in experiments. DLS values as well as the values of zeta potential were measured by Zetasizer (nano ZS, Malvern Instruments, USA). NP excitation/emission spectrums were verified by confocal laser scanning microscope (Zeiss 710 confocal microscope). All the results are presented in the Supporting Information (sections 13, 14 and 15). Non fluorescent TiO_2_ NPs of 10 nm size were characterized as described previously [49].

### Cell culture

Human lung adenocarcinoma (NCI-H292) cells were purchased from the American Type Culture Collection (Sigma-Aldrich, Saint Quentin Fallavier, France) and grown in RPMI 1640 (Roswell Park Memorial Institute) medium without phenol red (Life Technologies), containing 10% fetal calf serum (FCS, Life Technologies) and 1% glutaMAX^TM^ (Life Technologies), subsequently referred to as complete cell culture medium. The NCI-H292 cell line was derived from a lymph node metastasis of a pulmonary mucoepidermoid carcinoma. All experiments were performed with cells from passages 13 to 20. Cells were grown in T75-flasks (Costar, Sigma) as a monolayer. Exponentially growing cultures were maintained in a humidified atmosphere of 5% CO_2_ and 95% air at 37°C and were passaged twice weekly using 0.05% Trypsin-EDTA (Life Technologies) whose action was stopped with 10% FCS.

### Culture treatment

Depending on experiment, cells were seeded into cell culture plates (Costar, Sigma) and treated when they had reached 70-80% confluence. Before NP exposure, cells were rinsed with PBS to eliminate trace amounts of FCS. Treatments were performed in the absence of FCS as it has been found previously that the serum can form a protein corona modulating NP uptake and as the bronchial cells are not directly exposed to serum proteins conversely to internal organs. 50 nm-FITC-SiO_2_-NP stock (23.2 mg/mL in water) was vortexed shortly before making final dilution for the treatment. 100 nm-Por-SiO_2_-NPs stock suspension (5.25 mg/mL in water) was sonicated in an ultrasonic bath (Branson Cleaner, Ultrasonic, B200) at 20 W for 10 min then vortexed before making dilutions for treatment. TiO_2_ NPs stock suspension (2.56 mg/mL in water) was sonicated at 60 W (Ultrasonic processor, Bioblock Scientific) for 8 min. Cells were exposed for different times with either FITC labeled SiO_2_ NPs at 2.5 and 5 μg/cm^2^, porphyrine-labeled SiO_2_ NPs at 5, 15 and 25 μg/cm^2^ or non fluorescent TiO_2_ NPs at 20 and 40 μg/cm^2^.

For endocytic inhibition experiments, cells were pre-incubated for 30 min with different endocytic inhibitors at the following concentrations: chlorpromazine (CP) 25 μM, monodansylcadaverine (MDC) 75 μM, EIPA 75 μM, amiloride (A) 1.5 mM, nystatine (N) 75 μM and filipine (F) 4.5 μM (all from Sigma). Energy dependence experiments were performed by pre-incubating the cells at 4°C or with sodium azide (NaN_3_, 100 mM, Sigma) for 30 min prior to exposure to NPs. After these pre-incubations, NPs were added to cell cultures and incubated for 3.5 h, either in the presence of drugs or at 4°C.

### Flow cytometry (FCM) analysis

Cells were seeded in 12-well plates at 10,000 cells/cm^2^ in complete cell culture medium and incubated for 48 h before treatment. After treatment with 1.2 mL/well of 50 nm-FITC-SiO_2_-NPs at indicated concentrations, medium was removed, cultures were thoroughly washed with PBS and cells were harvested by trypsination whose action was stopped with 10% FCS. Shortly before FCM analysis cells were incubated with 0.11% Trypan Blue for 1 min in order to quench the FITC-fluorescent signal coming from NPs adsorbed to the cell surface. Cell-associated fluorescence was detected using a CyAn ADP LX (Dako Cytomation, Beckman Coulter, Villepinte, France) flow cytometer. Laser excitation and emission bandpass wavelengths were 488 nm and 575 ± 25nm respectively. Minimum of 10,000 cells was analyzed after exclusion of the cellular debris from the analysis by gating on the 575 nm Log versus FS area graph. The results are reported as the median of the distribution of cell fluorescence intensity obtained by analyzing 10,000 cells in the gate.

### Imaging flow cytometry analysis

Cells were seeded in 6-well plates at 10,000 cells/cm^2^ in complete cell culture medium and incubated for 48 h before treatment with 2.9 mL/well of 100 nm-Por-SiO_2_-NPs at indicated concentrations. At the end of the exposure to NPs, the media was removed, cells were thoroughly washed with PBS and cells were harvested. Cell suspension was centrifuged for 5 min at 200 *g* at 4°C and the cell pellet was resuspended in 500 μl of 4% para-formaldehyde (PFA, Santa Cruz Biotechnology Inc, Heidelberg, Germany). After 20 min of incubation in PFA, cells were rinsed three times with PBS and finally suspended in at least 50 μl of PBS. At least 2,500 cells were analyzed using Amnis ImageStream^x^ platform (Amnis, Proteigene, Saint Marcel, France) and Inspire^TM^ system software (Amnis). Camera magnification was 40x, 488 nm excitation laser was at 100 mW and 785 nm excitation laser was at 2.33 mW, except for non fluorescent NPs where it was set at 0.01 mW. The images were acquired with a normal depth of field, providing a cross-sectional image of the cell with a 4 μm depth of focus. A mask representing the whole cell was defined by the bright-field image, and an internal mask was defined by eroding the whole cell mask by 6 pixels (equivalent to 3 μm, as the size of 1 pixel is 0.5 μm) in order to eliminate the fluorescent signal coming from the NPs attached to the cell surface thus measuring only the internalized part. The results are analyzed by IDEAS software (Amnis),. Values of the internalization score, mean fluorescence intensity and mean side scatter intensity were calculated for at least 500 cells per sample.

### Confocal microscopy

Cells were seeded in 8 well Lab-Tek^TM^ chambered coverglasses (Nunc, Thermo Scientific, Dominique Dutscher, Brumath, France) at 40,000 cells/well in complete cell culture media. After treatment with 0.22 mL/well of NPs at indicated concentrations, cells were fixed in 4% PFA for 20 min at 25°C, rinsed 3 times with PBS and incubated for 10 min with NH_4_Cl (50 mM, Sigma) before permeabilization in 0.05% PBS-Tween20 (Sigma). To stain the actin filaments, cells were incubated for 30 min with FITC or TRITC-phalloïdine (0.9 nM in PBS,), then rinsed 4 times in PBS. Cells were mounted in Polyvinyl alcohol mounting medium with DABCO® (Sigma).

For immunolabelling experiments, cells were fixed with methanol at −20°C for at least 20 min, and rinsed three times with PBS. After permeabilization in 0.05% PBS-Tween20 and saturation in 0.01% PBS-Tween20–3% Bovine Serum Albumin (BSA, Sigma) cells were incubated for 60 min with goat polyclonal clathrin heavy chain antibody, C-20 (sc-6579, 1:50, Santa Cruz), goat polyclonal SNX5 D-18 (sc-10625, 1:50, Santa Cruz) or rabbit polyclonal caveolin1 N-20 (sc-894, 1:50, Santa Cruz) antibodies in 0.01% PBS-Tween20-3% BSA. Secondary anti goat antibodies Alexa fluor 488-IgG or 647-IgG (Life Technologies), anti rabbit antibodies Alexa fluor 488-IgG or 546-IgG (Life Technologies) were diluted in 0.01% PBS-Tween20-3% BSA at 1:400 and incubated for 45 min. Cell nuclei were stained with DAPI (4',6-Diamidino-2-Phenylindole, Dihydrochloride, Sigma, 0.25 μg/mL in PBS) for 1 min. Cells were examined under a Zeiss 710 confocal microscope using 63x objective (NA of 1.4) and a 1 to 1.5x zoom. The refractive index of immersion oil was 1.512. Considering optical laws the theoretical resolution was calculated and instrument settings adapted to obtain the best possible resolution in our images. Image treatment was done with Image J software (Image J 1.42 NIH, USA). The three-dimensional (3D) structure of the cells treated with NPs was reconstructed from corresponding confocal images using IMARIS software 7.5 (Bitplane). Pearson’s correlation coefficient was calculated using JACoP (Just Another Colocalization Plugin) for images with lateral resolution of 0.09 μm and axial resolution of 0.5μm.

### siRNA knockdown experiments

The expression of clathrin heavy chain (CHC) protein in NCI-H292 cells was knocked down by transfection with specific siRNA targeted against this protein (SI00299880, Qiagen, Courtaboeuf, France). Cells were seeded in 6-well plates at 15,000 cells/cm^2^ in complete culture medium. 24 h after seeding cells were treated with 100 nM siRNA-control or siRNA-clathrin heavy chain using 18 μl/well of Hiperfect transfection reagent (Qiagen) as indicated in the transfection protocol provided by the supplier. Cells were retransfected every 24 h until 72 h when transfection efficiency was monitored by confocal microscopy and Western blot. After treatment with siRNA, cells were incubated with NPs during 3.5 h.

### Statistical analysis

Every experiment was repeated at least twice with duplicates or triplicates of each condition. Data are represented as means ± SD or SEM and were analyzed on commercially available software SigmaStat (version 3.0, Systat software Inc, San Jose, California, USA) analysis of variance (one-way ANOVA) followed by Bonferroni post hoc test for multiple comparisons with p < 0.05 (two tailed) considered as significant.

## Competing interests

The authors declare that they have no competing interests.

## Authors’ contributions

SV contributed in study design, did the experimental work, analyzed data and wrote the manuscript. NB contributed in the creation of protocols and analysis of imaging flow cytometry data. VC did 3D reconstructions of confocal microscopic images and helped in colocalization study. SM and NR performed the synthesis of 50 nm-FITC-SiO_2_ NPs and provided TEM images of these NPs. FM critically reviewed the manuscript and gave intellectual input. SB and ABS contributed in the study design, analyzed data, coordinated the work and wrote the manuscript. All the authors have read and approved final manuscript.

## Supplementary Material

Additional file 1 **Supporting Figure S1.** Cell viability assay of NCI-H292 cells treated with NPs. **Supporting Figure S2**. Detection of DioC green fluorescence in viable cells in presence or not of Trypan Blue by FCM. **Supporting Figure S3**. Interaction of 100 nm-Por-SiO2 NPs with the cells at 4°C. Calculation of Internalization Score. **Supporting Figure S4**. Uptake of 50 nm-FITC-SiO2 by NCI-H292 cells studied by imaging flow cytometry. **Supporting Table 1**. Mechanism of action of pharmacological inhibitors of main endocytotic pathways. **Supporting Figure S5**. Cell viability assay after treatment with different pharmacological inhibitors. **Supporting Figure S6**. Confocal images of cells after treatment with pharmacological inhibitors. **Supporting Figure S7**. Confocal images of the cells after treatment with siRNA. **Supporting Figure S8**. Depletion of the expression of clathrin heavy chain (CHC) in siRNA-CHC transfected cells. **Supporting Figure S9**. NPs localization in the lysosomes. **Supporting Figures S10**. Cell viability assay of NCI-H292 cells treated with NPs. NP synthesis. **Supplementary Figure S11**. Transmission electron microscopy of SiO2 NPs in culture media. **Supplementary Table 2**. Physico-chemical characteristics of Silica NPs. **Supplementary Figure S12**. Fluorescence spectra of SiO2 NPs. Click here for file
