# Supplementary material for: Deciphering the mechanisms of cellular uptake of engineered nanoparticles by accurate evaluation of internalization using imaging flow cytometry
Source: Part Fibre Toxicol. 2013 Feb 6;10:2. doi: 10.1186/1743-8977-10-2 (PMC3599262; doi:10.1186/1743-8977-10-2)
Supplement: Additional file 1 — Supporting Figure S1. Cell viability assay of NCI-H292 cells treated with NPs. Supporting Figure S2. Detection of DioC green fluorescence in viable cells in presence or not of Trypan Blue by FCM. Supporting Figure S3. Interaction of 100 nm-Por-SiO2 NPs with the cells at 4°C. Calculation of Internalization Score. Supporting Figure S4. Uptake of 50 nm-FITC-SiO2 by NCI-H292 cells studied by imaging flow cytometry. Supporting Table 1. Mechanism of action of pharmacological inhibitors of main endocytotic pathways. Supporting Figure S5. Cell viability assay after treatment with different pharmacological inhibitors. Supporting Figure S6. Confocal images of cells after treatment with pharmacological inhibitors. Supporting Figure S7. Confocal images of the cells after treatment with siRNA. Supporting Figure S8. Depletion of the expression of clathrin heavy chain (CHC) in siRNA-CHC transfected cells. Supporting Figure S9. NPs localization in the lysosomes. Supporting Figures S10. Cell viability assay of NCI-H292 cells treated with NPs. NP synthesis. Supplementary Figure S11. Transmission electron microscopy of SiO2 NPs in culture media. Supplementary Table 2. Physico-chemical characteristics of Silica NPs. Supplementary Figure S12. Fluorescence spectra of SiO2 NPs. [file 1743-8977-10-2-S1.pdf]

Deciphering the mechanisms of cellular uptake of engineered nanoparticles by accurate evaluation of internalization using imaging flow cytometry

Sandra Vranic[1§], Nicole Boggetto[2], Vincent Contremoulins[2], Stéphane Mornet[3], Nora Reinhardt[3], Francelyne Marano[1], Armelle Baeza- Squiban[1]*, and Sonja Boland[1]*

Supporting information:

Outline

1. Cytotoxicity of SiO_2 nanoparticles (NPs)

2. Quenching of intracellular FITC fluorescence with Trypan Blue

3. Confocal images of the cells treated with 100 nm-Por-SiO_2 NPs at 4°C

4. Calculation of Internalization Score

5. Uptake of 50 nm-FITC-SiO_2 NPs studied by Imagestream

6. Mechanism of action of pharmacological inhibitors

7. Cytotoxicity of pharmacological inhibitors

8. Preservation of actin filaments after treatment with pharmacological inhibitors

9. Depletion of Clathrin Heavy Chain by siRNA

10. NP localization in lysosomes

11. Cytotoxicity of TiO_2 NPs

12. NP synthesis

13. Transmission Electron Microscopy (TEM)

14. Dynamic Light Scattering (DLS) analysis

15. Fluorescence spectra

1. Cytotoxicity of SiO_2 nanoparticles (NPs)

To determine non cytotoxic concentrations of NPs to be used for experiments, the metabolic activity of the cells was assessed by the WST-1 assay. Cells were seeded in 96-well plates at 10,000 cells per well in complete culture medium and incubated for 48 h before treatment with 100 µl of NPs at concentrations from 2.5 to 80 µg/cm² for 24 h. Metabolic activity was assessed using the WST-1 cell proliferation reagent (Roche, Meylan, France) according to the manufacturer's recommendations. After 4 h of incubation with cells, supernatants were transferred to a fresh plate in order to decrease the potential interference of NPs during the absorbance measurement. The absorbance was measured by dual wavelength spectrophotometry

at 450 and 630 nm using a microplate reader. Results are shown in Supporting Figure S1A. for 50 nm-FITC-SiO_2 NPs and S1B. for 100 nm-Por-SiO_2.

A significant decrease of metabolic activity was observed from 40 µg/cm² (50% of metabolic activity compared to control) for 50 nm-FITC-SiO_2 NPs while 100 nm-Por-SiO_2 decreased metabolic activity by 20% compared to non treated cells at 80 µg/cm^2 (Supporting Figures S1A. and S1B.).

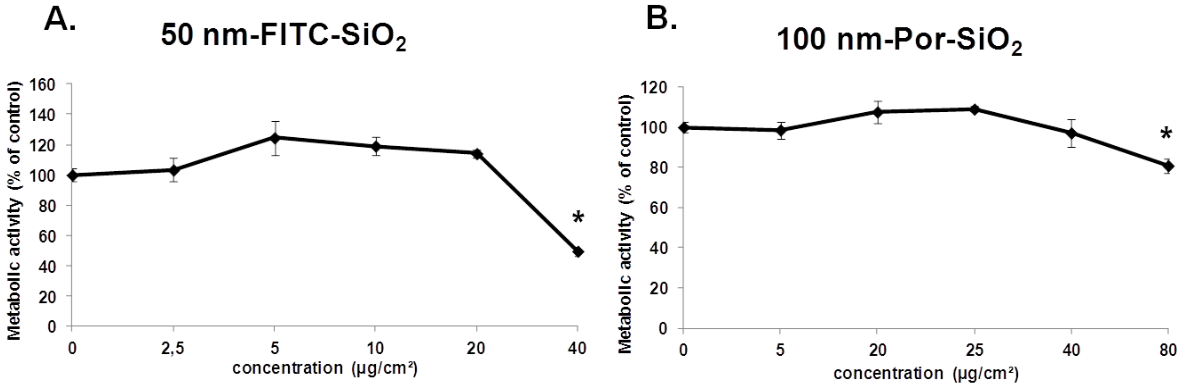

Supporting Figure S1. Cell viability assay of NCI-H292 cells treated with NPs. Cells were exposed for 24 h to SiO_2 NPs of 50 nm (A.) and 100 nm (B.) size at different concentrations. Metabolic activity was assessed by the WST-1 assay. The data were normalized to control values (no NP exposure) and expressed as mean ± SEM, $n = 6$ each. Data were analyzed by ANOVA, followed by Bonferroni post hoc test. * are indicating that there is a statistical difference compared to the control, $p < 0.05$.

2. Quenching of intracellular FITC fluorescence with Trypan Blue

Trypan Blue (TB) is a vital stain that enters only dead cells. When incubated with living cells it stays at the cell surface and should not be able to quench the fluorescence that is located inside

the cells. We assessed the efficiency of TB to quench only the fluorescence that is in direct contact by comparing the Median Fluorescence Intensity (MFI) of cells stained with 3,3′-Dihexyloxacarbocyanine iodide ($DiOC_6$ (3), Sigma), a mitochondrial marker, with and without TB. Cells were seeded in 12-well plates in complete cell culture medium at 10,000 cells/cm² for 48 h and then washed with Phosphate Buffered Saline (PBS, Life Technologies) and harvested by incubation with 0.05% Trypsin-EDTA for 10 min whose action was stopped by 10% FCS. The cell suspension was centrifuged for 5 min at 200 *g* then resuspended in media containing 1nM $DiOC_6$ (3) and incubated at 37°C in the dark for 30 min. Shortly before FCM analysis, cells were incubated with 0.11% Trypan Blue (Sigma) for 1 min. Cell-associated fluorescence was detected using a CyAn ADP LX (Dako Cytomation, Beckman Coulter, Villepinte, France) flow cytometer as described in methods section for 50 nm-FITC- SiO_2-NP uptake quantification.

Intracellular fluorescence remains the same when the cells were treated with 0.11% TB. (Supporting Figure S2).

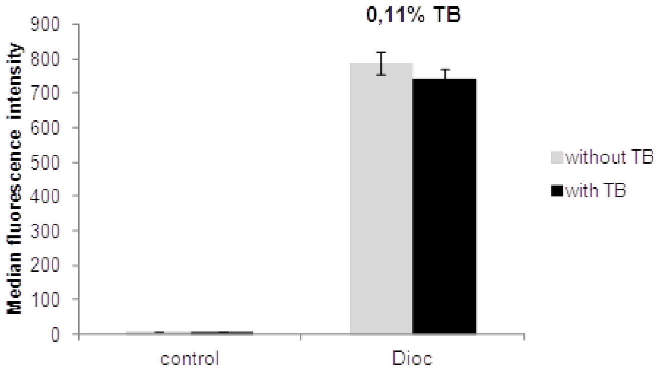

Supporting Figure S2. Detection of DioC green fluorescence in viable cells in presence or not of Trypan Blue by FCM. Cells were incubated for 30 min with DioC. Shortly before FCM

analysis, cells were incubated or not with 0.11% TB. MFI is determined for 10,000 cells. The data are expressed as mean ± SD, *n* = 3 each.

3. Confocal images of the cells treated with 100 nm-Por-SiO_2 NPs at 4°C

Cells were treated with 100 nm-Por-SiO_2 NPs for 4 h at 25 µg/cm² at 4°C in order to block the active as well as passive diffusion of NPs inside the cells and to verify the adsorption of NPs on the cellular membrane. Staining with phalloidin has been performed as described in the method section. After the treatment NPs were observed exclusively outside the cells and as a layer firmly attached to the cellular surface (Supporting Figure S3).

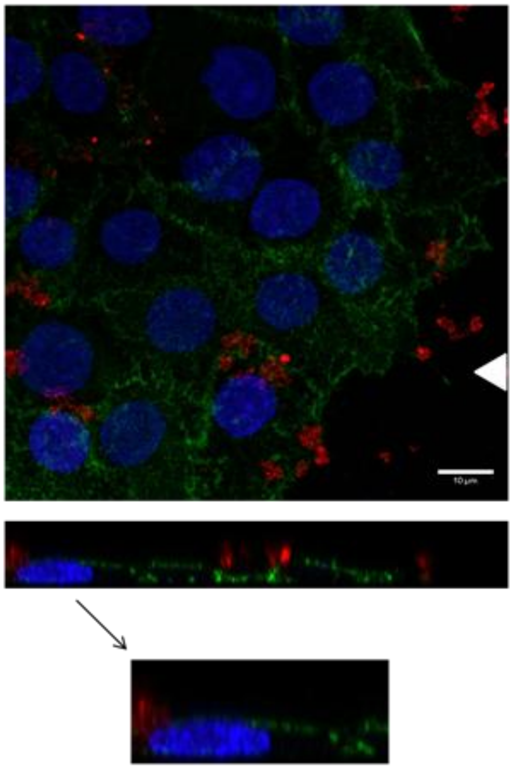

Supporting Figure S3. Interaction of 100 nm-Por-SiO_2 NPs with the cells at 4°C. Confocal image of the cells exposed to 100 nm-Por-SiO_2 NPs for 4 h at 25 µg/cm². The upper image

corresponds to the projection of all images acquired in the stack. Corresponding lower image represents x,z-slice of the section indicated with the white arrow. The insert shows one selected representative cell. Staining of the cells is as follows: Blue - DAPI-stained nuclei, Green - actin filaments staining, Red - Porphyrin-labelled SiO_2 particles. The scale bar shows 10 µm.

4. Calculation of Internalization Score

The Internalization Score (IS) is defined as the ratio of intensity inside the cell to the intensity of the entire cell. For robustness, only the upper quartile of pixels (based on intensity) inside the cell and in the membrane are considered for computing the ratio. Also, in order to provide invariance to cell size, the mean of the upper quartile pixel intensities is used to compute the ratio. The ratio is then mapped onto a log scale to increase the dynamic range. The thickness of the membrane (in pixels) determines which pixels are used to define the boundary and the membrane portions of the cell. The 'internal' mask is based on the brightfield image that covers the inside of the cell, the thickness of the membrane in pixels and the fluorescent channel of interest.

The cell is divided into 2 regions: External (B) and internal (I). The internal region is defined with the mask. The external region is determined by: 1. Dilating the internal mask by the membrane thickness. 2. Combining 1 with the object mask of the channel of interest. 3. External region equals mask 2 and not the internal mask. Next, the mean intensity of the upper quartile of the pixels in each region is determined. The Internalization Score (IS) is then computed as follows:

$IS = \log(a/1-a)$, where $a = (m_I/ m_I + m_B)(p_I/p_B)$

m_I = Mean intensity of upper quartile pixels in I, m_B = Mean intensity of upper quartile pixels in B,

p_I = Peak intensity of upper quartile pixels in I, p_B = Peak intensity of upper quartile pixels in B

5. Uptake of 50 nm-FITC-SiO_2 NPs studied by Imagestream

In order to compare the Imagestream technique with TB quenching using classical flow cytometry we analyzed values of MFI of the total cell and after applying the mask eroded for 6 pixels to compare them with results obtained with and without TB of Figure 2. Cells were seeded in 6-well plates at 10,000 cells/cm² in complete cell culture medium and incubated for 48 h before treatment with 2.9 mL/well of 50 nm-FITC-SiO_2-NPs at 2.5 and 5µg/cm² for 4 and 24 h. Cells were prepared for analysis as described in the method section. Camera magnification was 40 x, a 488 nm excitation laser at 20 mW and a 785 nm excitation laser at 2.33 mW were used. The images were acquired with a normal depth of field, providing a cross-sectional image of the cell with a 4 µm depth of focus. We observed that the values inside the eroded mask were lower than the ones for the total cell for both tested concentrations and had very similar tendency as the values obtained by flow cytometry before and after adding TB (Supporting Figure S4).

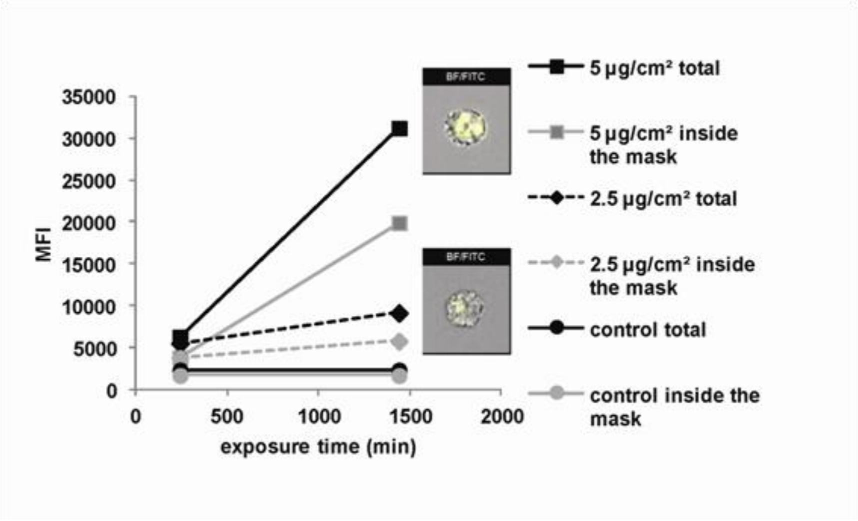

Supporting Figure S4. Uptake of 50 nm-FITC-SiO_2 by NCI-H292 cells studied by imaging flow cytometry. Cells were treated with 2.5 and 5 µg/cm² of 50 nm-FITC-SiO_2 NPs for 4 and 24 h, then harvested and fixed in 4% PFA. Mean fluorescence intensity (MFI) was calculated for the entire cell and inside the mask eroded for 3 µm representing the inside of the cell using Amnis IDEAS software. Representative images of the cells treated with 2.5 and 5 µg/cm² of 50 nm-FITC-SiO_2 NPs for 24 h, captured by the Amnis ImageStream^X Flow Cytometer, show the fluorescence emissions after excitation at 488 nm merged with the brightfield images of the cells.

6. Mechanism of action of pharmacological inhibitors

Three endocytotic pathways were intensively investigated: clathrin dependent endocytosis, caveolae dependent endocytosis and macropinocytosis.[1] For each of the three main endocytotic pathways we used two pharmacological inhibitors considered as specific. The mechanism of action of these inhibitors is listed in the Supporting Table 1.

Inhibitor	Mechanism of action	Endocytotic pathway
Chlorpromazine	Loss of clathrin and the AP2 adaptor complex from the cell surface and their artificial assembly on endosomal membranes [2, 3]	Clathrin dependent endocytosis
Monodansylcadaverine	Stabilization of clathrin-coated pits by the drug [4] but observed only in cell-free systems [5]	
EIPA	Blockage of the activity of the Na+/H+ exchanger isoform 1 when used in micromolar (EIPA, [6, 7]) and milimolar concentrations (Amiloride, [8]).	Macropinocytosis/phagocytosis
Amiloride		
Nystatine	Profound distortion of the structure and functions of the cholesterol-rich membrane domain, including aberrations in the caveolar shape [9], dispersion of GPI-anchored proteins from these structures [10], as well as the inhibition of lipid raft ligands internalization [11].	Caveolae-dependent endocytosis
Filipine		

Supporting Table 1. Mechanism of action of pharmacological inhibitors of main endocytotic pathways.

7. Cytotoxicity of metabolic inhibitors

The concentration of inhibitors used in experiments was chosen after determination of non cytotoxic concentrations by the WST-1 test and propidium iodide (PI) staining. For metabolic activity assays cells were seeded in 96-well plates at 10,000 cells per well in complete culture medium and incubated for 48 h before treatment with 100 µl of inhibitors for 4 h at the

concentrations used in experiments: chlorpromazine (CP) 25 µM, monodansylcadaverine (MDC) 75 µM, EIPA (E) 75 µM, amiloride (A) 1.5 mM, nystatine (N) 75 µM, filipine (F) 4.5 µM and NaN_3 100 mM. After treatment, metabolic activity of the cells was evaluated by WST-1 test as already described. For estimation of the percentage of dead cells by PI staining using flow cytometry, cells were seeded in 12-well plates in complete medium at 10,000 cells/cm² for 48 h before treatment with inhibitors at concentrations used in experiments. After treatment with different metabolic inhibitors, cells were washed with PBS and harvested. Shortly before analysis, cells were incubated with PI at a final concentration of 1.8 µg/mL. Cell-associated fluorescence was detected using a CyAn ADP LX (Dako Cytomation, Beckman Coulter, Villepinte, France) flow cytometer. Laser excitation and emission bandpass wavelengths were 488 nm and 530 ± 40nm respectively. A minimum of 10,000 cells were analyzed after exclusion of the cellular debris from the analysis by gating on the 530 nm Log versus FS area graph.

The WST-1 test revealed that the metabolic activity of the cells was not impaired (Supporting Figure S5A.) while PI staining showed that the number of dead cells was not significantly different from the one obtained in non treated cells (Supporting Figure S5B.).

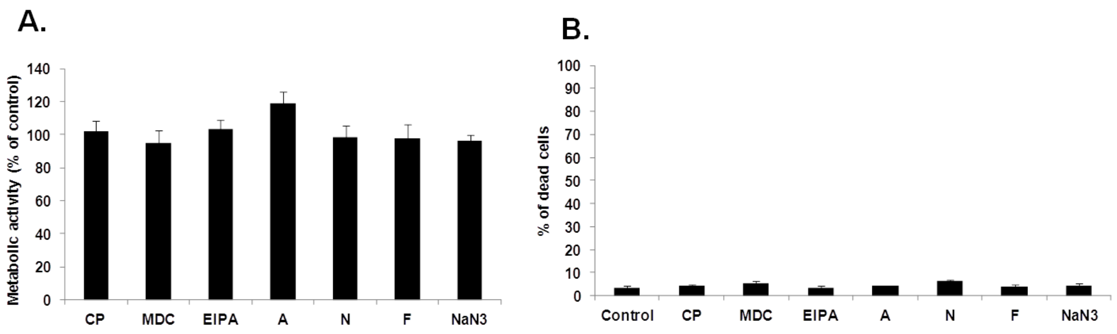

Supporting Figure S5. Cell viability assay after treatment with different pharmacological inhibitors. A. Cells were treated with metabolic inhibitors for 4 h at concentrations used in

experiments (CP – 25 μM, MDC – 75 μM, EIPA – 75 μM, A - 1.5 mM, N – 75 μM, F - 4.5 μM, NaN_3 – 100 mM). The metabolic activity of the cells after treatment was assessed by the WST-1 assay. The data were normalized to control values (no exposure to inhibitors) and expressed as mean ± SEM, *n* = 6 each. B. % of dead cells was determined by quantification of PI positive cells by FCM measurements of 10,000 cells. The data are expressed as mean ± SD, *n* = 3 in two independent experiments.

8. Preservation of actin filaments after treatment with pharmacological inhibitors

The effect of pharmacological inhibitors on the actin filaments was verified by confocal microscopy. Cells were treated with inhibitors for 4 h at concentrations used in experiments before staining of actin filaments with phalloidin. Experiments have been performed as described in the method section.

Confocal microscopy revealed that actin filaments were intact after the treatment with inhibitors (Supporting Figure S6).

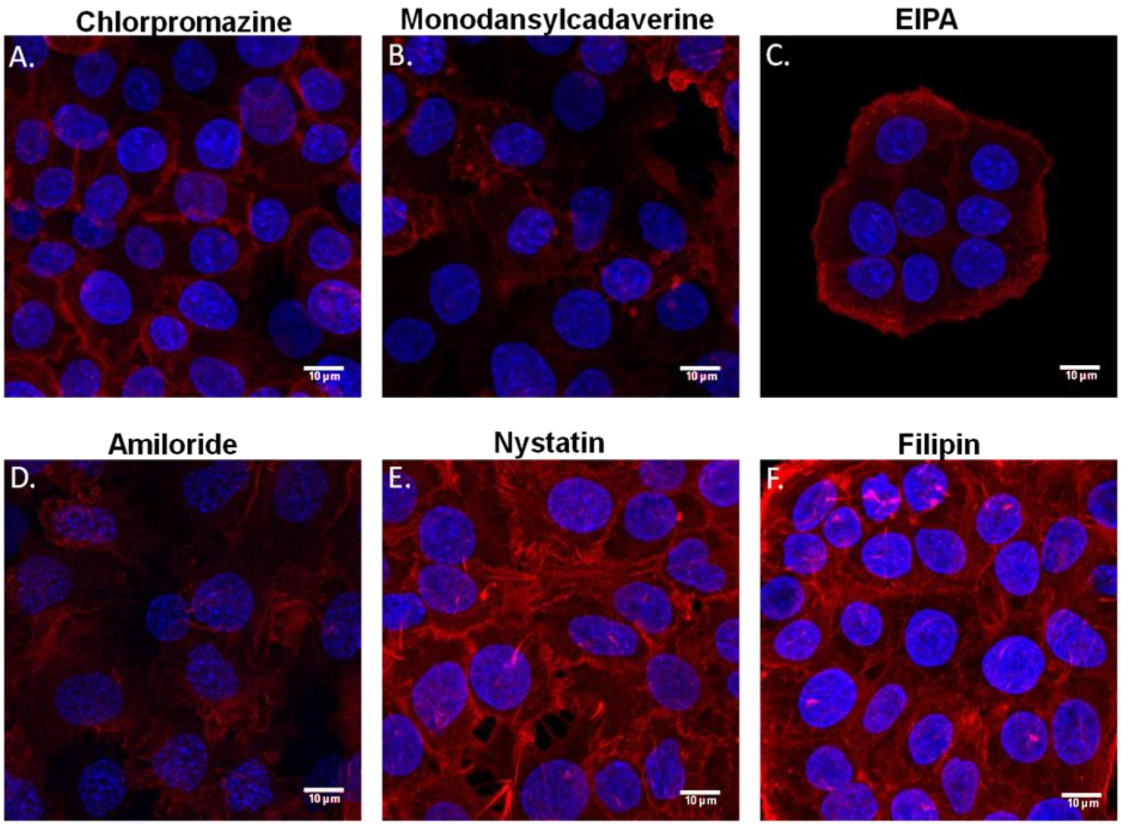

Supporting Figure S6. Confocal images of cells after treatment with pharmacological inhibitors. Cells were exposed for 4h to pharmacological inhibitors: CP – 25 µM (A.), MDC – 75 µM (B.), EIPA – 75 µM (C.), Amiloride – 1.5 mM (D.), Nystatin – 75 µM (E) and Filipin – 4.5 µM (F). Images correspond to the projection of all images acquired in the stack. Staining of the cells is as follows: Blue - DAPI-stained nuclei, Red –actin filaments staining. Scale bars show 10 µm.

9. Depletion of Clathrin Heavy Chain by siRNA

In order to deplete expression of clathrin heavy chain (CHC) protein, cells were transfected either with 100 nM siRNA-control (CTRL, negative control) or siRNA-clathrin heavy chain

(CHC), using the Hiperfect transfection reagent (Qiagen) as indicated in the transfection protocol provided by the supplier. Transfections were repeated every 24 h until 72 h, when the expression of protein was verified. The efficiency of the siRNA-clathrin heavy chain was assessed by confocal microscopy after immunofluorescence staining with goat polyclonal clathrin heavy chain antibody, C-20 (sc-6579, 1:50, Santa Cruz) (Supporting Figure S7) and by Western Blot analysis (Supporting Figure S8).

Immunolabelling was performed as described in the method section. Comparing images of the cells treated with siRNA-clathrin heavy chain and siRNA-control, a clear difference in expression of CHC protein can be observed. In the cells treated with siRNA-clathrin heavy chain, immunolabelling of CHC protein was weaker than in the cells treated with siRNA-control (Supporting Figure S7).

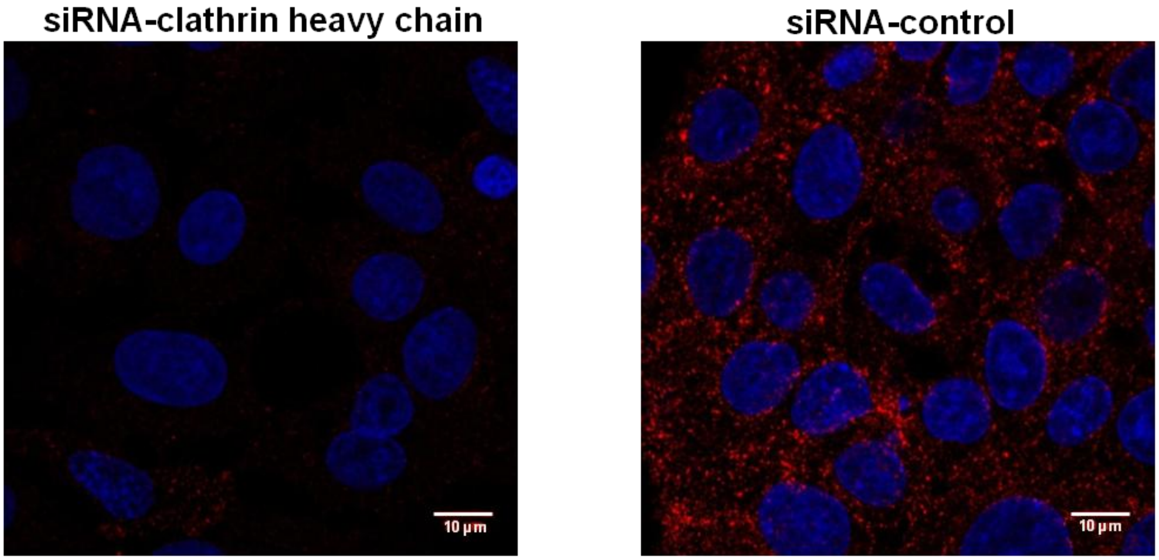

Supporting Figure S7. Confocal images of the cells after treatment with siRNA. Cells were treated either with 100 nM siRNA-clathrin heavy chain or siRNA-control for 72 h. Staining of

the cells is as follows: Blue - DAPI-stained nuclei, Red – CHC protein labelling. Images correspond to the projection of all images acquired in the stack. Scale bars show 10 µm.

For western blot analysis after washing with PBS, transfected cells were collected by trypsination. Cells were centrifuged for 5 min at 200 *g* and then lysed on ice for 30 min in Laemmli buffer (60 mM Tris-HCl, pH 6.8, 10% glycerol, 2% Sodium Dodecyl Sulfate, all from Sigma) and sonicated for 1 min at 60 W (Ultrasonic processor, Bioblock Scientific). Samples were centrifuged for 15 min, at 16 000 *g* at 4°C. The samples containing 4x SDS sample buffer (Bromophenol Blue 0.1%, DiThioThreitol 50 mM, Sodium Dodecyl Sulfate (SDS) 2%; Glycerol 35%, Tris-HCl 62.5 mM, all from Sigma) were boiled for 5 min at 95°C, and the proteins were separated by 7.5% SDS-PolyAcrylamide Gel Electrophoresis. Proteins were electrotransferred onto a nitrocellulose membrane for 1 h at 300 mA. The membrane was blocked by incubation with Tris-buffered saline/Tween-20 (TBS, Sigma) supplemented with 5% nonfat milk powder for 30 min. Antibodies were added and the membrane was incubated overnight at 4°C in TBS supplemented with 1% nonfat milk powder. Immunoblotting was performed using primary antibodies: goat polyclonal anti-clathrin heavy chain antibody (1:1000, sc-6579, Santa Cruz), mouse monoclonal anti-actin antibody (1:4000, sc-8432, Santa Cruz), followed by horseradish peroxidase-conjugated goat (1:1000, sc-2056, Santa Cruz) or mouse-specific secondary antibody (1:8000, A9044, Sigma). Development of peroxidase activity was performed with the Enhanced ChemiLuminescence detection kit (BioFX Laboratories, SurModics Inc., Euromedex, Mundolsheim, France). Quantification of the expression of the protein was performed by Multi Gauge software (FUJIFILM, Bois D'Arcy, France).

The protein extract from cells transfected with siRNA-clathrin heavy chain showed a clear lower immunoreactivity for CHC antibody compared to the extract from cells transfected with siRNA-control (Supporting Figure S8A.) Quantification of the protein expression after transfection (Supporting Figure S8B.) revealed 17% of the remaining expression in the siRNA-clathrin heavy chain transfected cells compared to the cells treated with siRNA-control that had 100% of the protein expression.

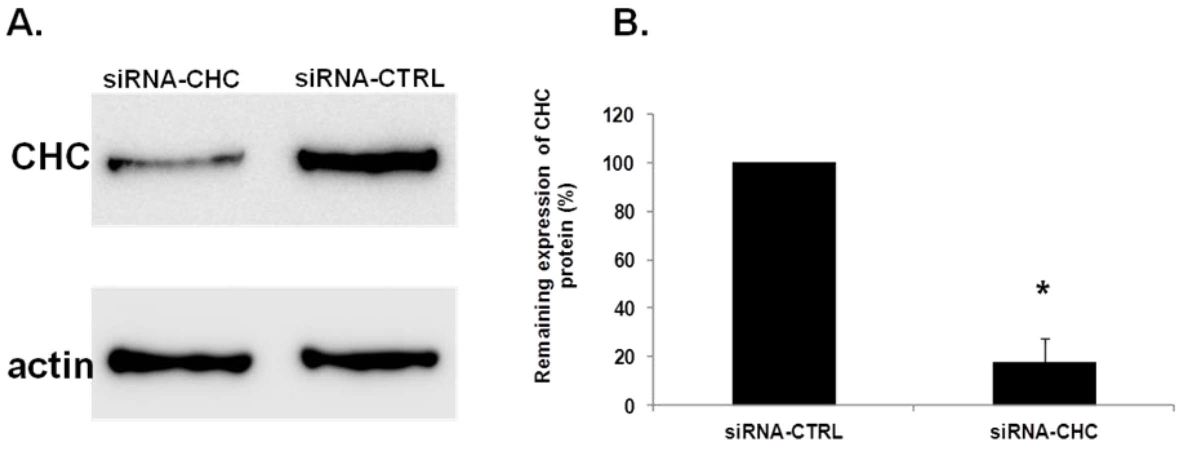

Supporting Figure S8. Depletion of the expression of clathrin heavy chain (CHC) in siRNA-CHC transfected cells. A. Western blot analysis of CHC depletion in siRNA-CHC treated cells. Lysates were prepared 72 h after transfection with siRNA-CHC or siRNA-control (CTRL). Actin is used as an internal control. B. Quantification of the expression of the protein after treatment with siRNA. SD is calculated for three independent experiments where expression of CHC protein in the siRNA-CTRL treated cells has been set as 100%. Data were analyzed by ANOVA, followed by Bonferroni post hoc test. * are indicating that there is a statistical difference compared to the control, $p < 0.05$.

10. NP localization in lysosomes

To investigate whether NPs were localized within lysosomes after internalization cells were treated with 50 nm-FITC-SiO_2 NPs and 100 nm-Por-SiO_2 NPs for 4 and 24 h and lysosomes were stained using the lysosomal marker LAMP. Fixation, permeabilization and saturation were performed as described in method section. Cells were incubated for 60 min with mouse monoclonal lysosome-associated membrane proteins (LAMP) antibody, H4A3 (sc-20011, 1:50, Santa Cruz), in 0.01% PBS-Tween-20-3% BSA. Secondary anti mouse antibody, Alexa fluor 488-IgG or 647-IgG (Life Technologies), were diluted in 0.01% PBS-Tween-20-3% BSA at 1:400 and incubated for 45 min. Cell nuclei were stained with DAPI (4, 6-Diamidino-2-Phenylindole, Dihydrochloride, Sigma, 0.25 µg/mL in PBS) for 1 min. Cells were examined under a Zeiss 710 confocal microscope using 63x objective and a 1.5 x zoom. Image treatment was done with Image J software (Image J 1.42 NIH, USA). SiO_2 NPs could be observed in lysosomes after 4 h of treatment (Supporting Figures S9A and S9C), and even more after 24 h (Supporting Figure S9B and S9D). Interestingly, all 100 nm-Por-SiO_2 NPs seem to be trapped in the lysosomes after 24 h.

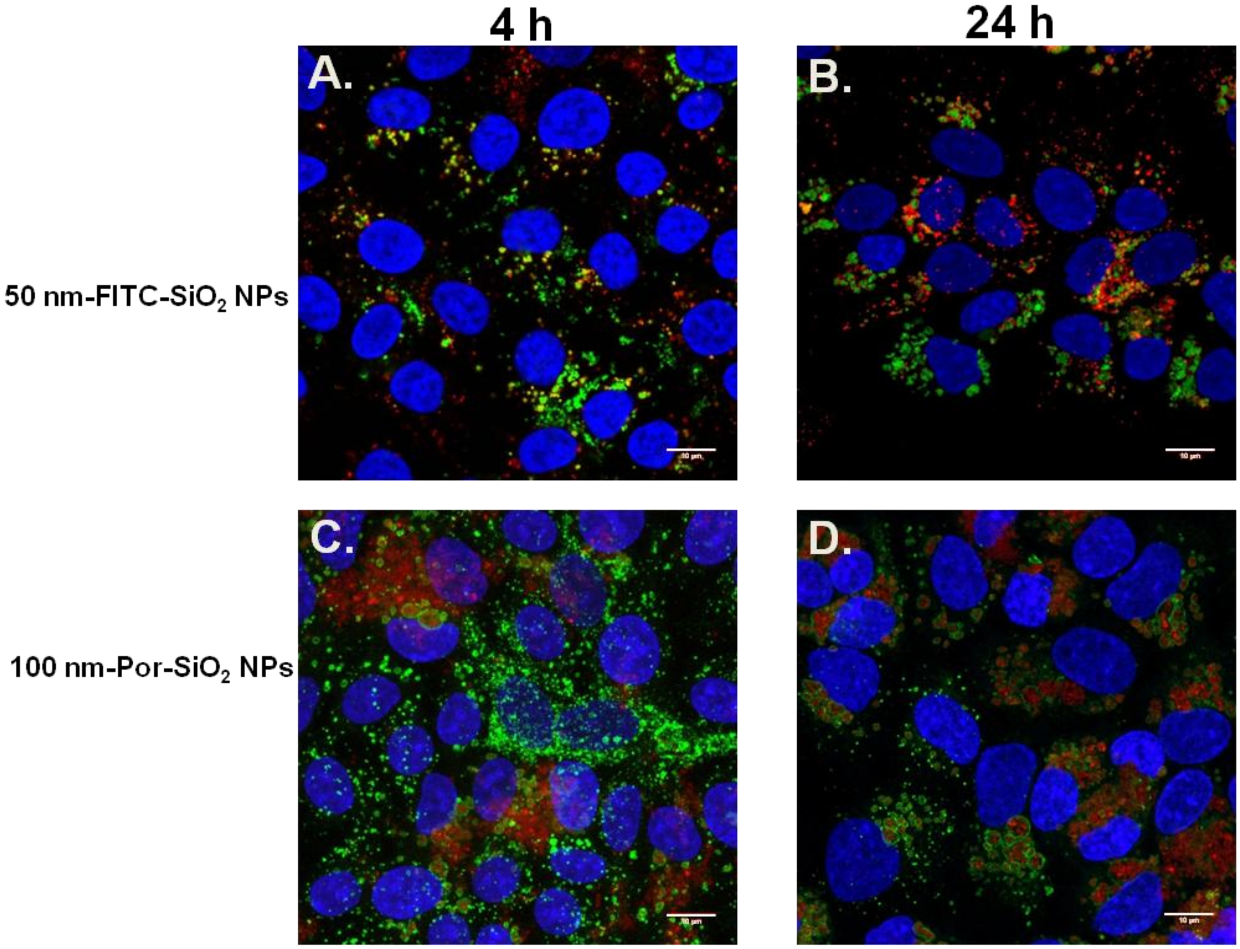

Supporting Figure S9. NPs localization in the lysosomes. Confocal images of the cells exposed to 50 nm-FITC-SiO_2 (A. and B.) and 100 nm-Por-SiO_2 NPs (C. and D.) for 4 (A. and C.) and 24 h (B. and D.) at 5 and 25 µg/cm² respectively. The images correspond to the projection of all images acquired in the stack. Staining of the cells is as follows: 50 nm-FITC-SiO_2 NPs, Blue - DAPI-stained nuclei, Red –LAMP labelling, Green - FITC-labelled SiO_2 NPs; 100 nm-porphyrine-SiO_2 NPs, Blue - DAPI-stained nuclei, Green – LAMP labelling, Red - Porphyrine-labelled SiO_2 particles. Scale bars show 10 µm.

11. Cytotoxicity of TiO_2 NPs

To determine non cytotoxic concentrations of TiO_2 NPs to be used in experiments, the metabolic activity of the cells was asessed by WST-1 assay. Cells were seeded in 96-well plates at 10,000 cells per well in complete cell culture medium and incubated for 48 h before treatment with 100 µl of NPs from 5 to 80 µg/cm² for 24 h. Metabolic activity was assessed as already described. TiO_2 NPs did not impair the metabolic activity of the treated cells at any concentration tested (Supporting Figure S10).

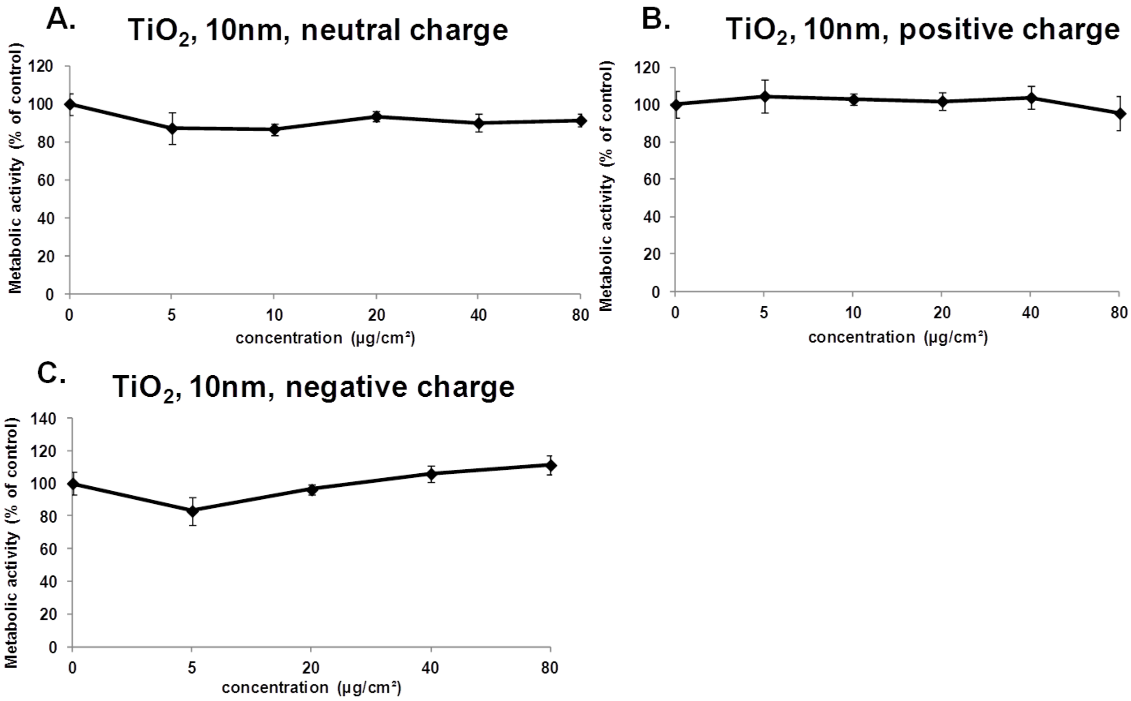

Supporting Figures S10. Cell viability assay of NCI-H292 cells treated with NPs. Cells were exposed for 24 h to different concentrations of TiO_2 NPs of neutral charge (A.), positive charge (B.) and negative charge (C.). Metabolic activity was assessed by the WST-1 assay. The data were normalized to control values (no NP exposure) and expressed as mean ± SEM, $n = 6$ each.

12. NP synthesis

50 nm-FITC-SiO_2-NPs were synthesized following a slightly modified method described by Van Blaaderen to obtain the expected size[12]. In a first step fluorescein isothiocyanate (FITC) was covalently attached to a coupling agent (3-aminopropy1) triethoxysilane (APS) by reaction of the amino group with isothiocyanate group. The reaction was performed in the dark to avoid photobleaching and under anhydrous conditions to prevent hydrolysis of APS. Typically, 5 mg of FITC was dissolved in 5 mL of 42.7 mM of APS in ethanol. After 12 h of stirring, the fluorescent silane was added to a 500 mL two-neck flask containing 250 mL ethanol, 5 mL tetraethoxysilane (TEOS), 7.6 mL ammonium hydroxide (28 %) and 10.9 mL water in 50°C oil bath. The reaction continued for 12 h in the dark under magnetic stirring. The as-prepared particles have an average diameter of about 30 nm. A seed-growth procedure was used to increase the NP size to 50 nm. The experiment is carried out as follows: the entire mixture was poured into a 1 L round bottom flask containing 500 mL ethanol, 160 mL water and 9.9 mL ammonium hydroxide (28%). Then 18.2 mL of TEOS was added twice spaced 12 h apart to prevent secondary nucleation. The resulting particles have an approximate average diameter of 50 nm measured from TEM images. After the synthesis, ammonia and ethanol were removed from the medium by rotary evaporation at 40°C. The fluorescent particles were extensively washed by centrifugation against ultrapure water (18 MΩ) at 13,000 *g* for 15 min until disappearance of fluorescence in the supernatant. Estimation of silica concentration in the dispersion was carried out by inductively coupled plasma optical emission spectrometry (ICP-OES) and gravimetric method.

13. Transmission Electron Microscopy (TEM)

NPs were characterized for their morphology by TEM (TEM, JEOL 1200 EXII (OXFORD LINK ISIS 300)). 50 nm-FITC-SiO_2-NPs and 100 nm-Por-SiO_2-NPs diluted in cell culture medium were predominantly observed in aggregates of at least 300 nm and 1 µm respectively (Supplementary Figure S11).

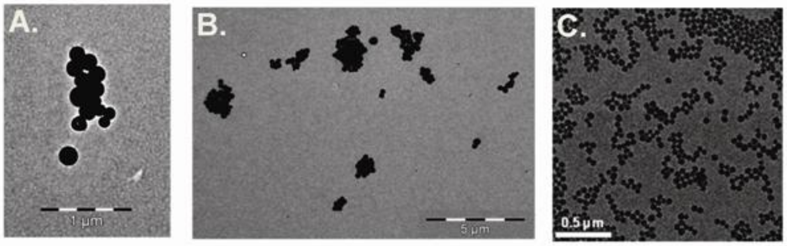

Supplementary Figure S11. Transmission electron microscopy of SiO_2 NPs in culture media. A and B. TEM of 100 nm-SiO_2-NPs labeled with 5,10,15,20-Tetrakis-(1-methyl-4pyridino) porphyrine tetra(toluene-4-sulfonate). The scale bar shows 1 and 5 µm in the panels A. and B. respectively. C. TEM of 50 nm-SiO_2-NPs labeled with fluorescein isothiocyanate. The scale bar shows 500 nm.

14. Dynamic Light Scattering (DLS) analysis

NPs were characterized for their hydrodynamic diameter and zeta potential whilst suspended in RPMI. DLS and zeta potential values were measured by a Zetasizer (nano ZS, Malvern Instruments, USA, Supplementary Table 2). Distribution curves show aggregation for both NPs. 50 nm-FITC-SiO_2-NPs made small aggregates (242.7 nm) while 100 nm-Por-SiO_2-NPs made

larger ones. 50 nm-FITC-SiO_2-NPs have a negative surface charge in RPMI as well as 100 nm-Por-SiO_2-NPs.

	Size	Zeta potential (mV)	Hydrodynamic diameter (nm)
	(ø) nm	RPMI	RPMI
SiO_2	50	-9.8	242.7
SiO_2	100	-20.8	1216

Supplementary Table 2. Physico-chemical characteristics of Silica NPs.

15. Fluorescence spectra

As NPs were coupled to different fluorochromes, their emission wavelengths were verified by confocal microscopy (Zeiss 710 confocal microscope). 50 nm-FITC-SiO_2-NPs excited at 488 nm had the peak of emission obtained at 522 nm, while for 100 nm-Por-SiO_2-NPs excited at 405 nm (maximum excitation is at 422 nm) the maximum emission was collected at 666 nm (Supplementary Figures S12A and S12B).

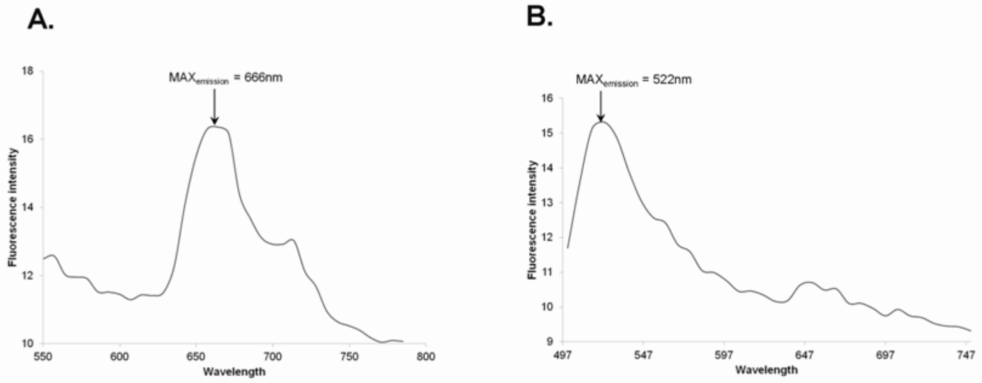

Supplementary Figure S12. Fluorescence spectra of SiO_2 NPs. A. Emission spectra of SiO_2 NPs labeled with 5,10,15,20-Tetrakis-(1-methyl-4pyridino) porphyrin tetra(toluene-4-sulfonate) obtained by Zeiss confocal microscope 710 after excitation at 405 nm. B. Emission spectra of SiO_2 NPs labeled with fluorescein isothiocyanate obtained by Zeiss confocal microscope 710 after excitation at 488 nm.

1. Conner, S. D.; Schmid, S. L., Regulated portals of entry into the cell. *Nature* 2003, 422, 37-44.

2. Yao, D.; Ehrlich, M.; Henis, Y. I.; Leof, E. B., Transforming growth factor-beta receptors interact with AP2 by direct binding to beta2 subunit. *Mol Biol Cell* 2002, 13, 4001-12.

3. Huotari, V.; Vaaraniemi, J.; Lehto, V. P.; Eskelinen, S., Regulation of the disassembly/assembly of the membrane skeleton in Madin-Darby canine kidney cells. *J Cell Physiol* 1996, 167, 121-30.

4. Wang, J.; Liu, X. J., A G protein-coupled receptor kinase induces Xenopus oocyte maturation. *J Biol Chem* 2003, 278, 15809-14.

5. Nandi, P. K.; Van Jaarsveld, P. P.; Lippoldt, R. E.; Edelhoch, H., Effect of basic compounds on the polymerization of clathrin. *Biochemistry* 1981, 20, 6706-10.

6. Maréchal, V.; Prevost, M. C.; Petit, C.; Perret, E.; Heard, J. M.; Schwartz, O., Human immunodeficiency virus type 1 entry into macrophages mediated by macropinocytosis. *J Virol* 2001, 75, 11166-77.

7. Nakase, I.; Niwa, M.; Takeuchi, T.; Sonomura, K.; Kawabata, N.; Koike, Y.; Takehashi, M.; Tanaka, S.; Ueda, K.; Simpson, J. C.; Jones, A. T.; Sugiura, Y.; Futaki, S., Cellular uptake of arginine-rich peptides: roles for macropinocytosis and actin rearrangement. *Mol Ther* 2004, 10, 1011-22.

8. West, M. A.; Bretscher, M. S.; Watts, C., Distinct endocytotic pathways in epidermal growth factor-stimulated human carcinoma A431 cells. *J Cell Biol* 1989, 109, 2731-9.

9. Ros-Baro, A.; Lopez-Iglesias, C.; Peiro, S.; Bellido, D.; Palacin, M.; Zorzano, A.; Camps, M., Lipid rafts are required for GLUT4 internalization in adipose cells. *Proc Natl Acad Sci U S A* 2001, 98, 12050-5.

10. Rothberg, K. G.; Ying, Y. S.; Kamen, B. A.; Anderson, R. G., Cholesterol controls the clustering of the glycophospholipid-anchored membrane receptor for 5-methyltetrahydrofolate. *J Cell Biol* 1990, 111, 2931-8.

11. Orlandi, P. A.; Fishman, P. H., Filipin-dependent inhibition of cholera toxin: evidence for toxin internalization and activation through caveolae-like domains. *J Cell Biol* 1998, 141, 905-15.

12. Blaaderen, A. v.; Vrij, A., Synthesis and Characterization of Colloidal Dispersions of Fluorescent, Monodisperse Silica Spheres. Langmuir, 1992; Vol. 8, pp 2921-2931.
